# Risk factors for inflammatory bowel disease: an umbrella review

**DOI:** 10.3389/fcimb.2024.1410506

**Published:** 2025-01-24

**Authors:** Tingping Wu, Honghui Cheng, Jiamei Zhuang, Xianhua Liu, Zichen Ouyang, Rui Qian

**Affiliations:** ^1^ Shenzhen Bao'an Chinese Medicine Hospital, The Seventh Clinical College of Guangzhou University of Chinese Medicine, Shenzhen, Guangdong, China; ^2^ The Fourth Clinical Medical College of Guangzhou University of Chinese Medicine, Shenzhen, Guangdong, China

**Keywords:** inflammatory bowel disease, risk factor, umbrella review, meta-analysis, IBD

## Abstract

**Introduction:**

Inflammatory bowel disease (IBD) represents a cluster of chronic idiopathic inflammatory disorders situated at the nexus of intricate interplays. The primary aim of the present investigation is to perform an umbrella review of metaanalyses, systematically offering a comprehensive overview of the evidence concerning risk factors for IBD.

**Methods:**

To achieve this, we searched reputable databases, including PubMed, Embase, Web of Science, and the Cochrane Database of Systematic Reviews, from inception through April 2023. Two authors independently assessed the methodological quality of each metaanalysis using the AMSTAR tool and adhered to evidence classification criteria.

**Results:**

In total, we extracted 191 unique risk factors in meta-analyses, including 92 significantly associated risk factors. The top ten risk factors were human cytomegalovirus (HCMV) infection, IBD family history, periodontal disease, poliomyelitis, campylobacter species infection, hidradenitis suppurativa, psoriasis, use of proton pump inhibitors, chronic obstructive pulmonary disease, and western dietary pattern.

**Discussion:**

In conclusion, this umbrella review extracted 62 risk factors and 30 protective factors, most of which were related to underlying diseases, personal lifestyle and environmental factors. The findings in this paper help to develop better prevention and treatment measures to reduce the incidence of IBD, delay its progression, and reduce the burden of IBD-related disease worldwide.

**Systematic review registration:**

https://www.crd.york.ac.uk/PROSPERO/, identifier CRD42023417175.

## 1 Introduction

Inflammatory bowel disease (IBD) represents a collection of chronic idiopathic inflammatory disorders situated at the nexus of intricate interplays involving genetics, environmental factors, and the gut microbiota. This encompasses Crohn’s disease (CD) and ulcerative colitis (UC) (Ananthakrishnan, 2015; Piovani et al., 2019). IBD has an influence on about 1.5 million Americans, 2.2 million people in Europe (Cosnes et al., 2011; Molodecky et al., 2012). Besides, statistically, the incidence of IBD in South America, Africa and Asia has continued to rise over the past few decades (Ng et al., 2017). Incidence of IBD is rising in populations previously regarded as “low risk”, such as Japan and India (Ananthakrishnan, 2015). The prolonged duration of IBD results in a notable treatment burden, frequent hospitalizations, surgical interventions, and a substantial influence on the quality of life, economic productivity, and social functioning of affected individuals (Cosnes et al., 2011; Peery et al., 2019). Given the increasing incidence and severe disease burden of IBD and the high cost of treatment, it is necessary to better understand the potential risk factors of IBD and to adopt effective prevention strategies.

The etiology of IBD is acknowledged to be multifaceted, with the initiation and progression of IBD being influenced by a combination of genetic and environmental factors (Ng et al., 2013; de Lange et al., 2017). In recent decades, there has been an acknowledgment of the heritability of IBD. Notably, in 2001, the identification of the inaugural gene associated with Crohn’s disease marked a significant milestone in this understanding (Hugot et al., 2001; Ogura et al., 2001). Subsequent researches observed 163 risk alleles associated with IBD in white populations (Jostins et al., 2012). Besides, it is recognized that genetic factors are not the only factors contributing to IBD, but act in combined with environmental factors (Ananthakrishnan, 2015). A large number of meta-analyses have identified several risk factors for IBD, mainly including smoking, urban living, appendectomy, tonsillectomy, antibiotic use, oral contraceptive use, consumption of soft drinks, vitamin D deficiency, depression, obesity, and psoriasis (Fu et al., 2018; Piovani et al., 2019; Rahmani et al., 2019; Luo et al., 2022; Narula et al., 2023). In addition, several protective factors of IBD were also identified in meta-analyses, such as exercise, tea consumption, high levels of folate, and high levels of vitamin D (Piovani et al., 2019).

Despite numerous meta-analyses of observational studies have evaluated a range of risk factors of IBD during recent years, drawbacks in the research design, different assessments of exposure factors, and inconsistent outcomes make it difficult to draw definitive conclusions (Heikkilä et al., 2014; Timm et al., 2014; Ge et al., 2015; Liu et al., 2015; Lu et al., 2015; Pineton de Chambrun et al., 2015; Cholapranee and Ananthakrishnan, 2016; Castaño-Rodríguez et al., 2017; Cipriani et al., 2017; Lv et al., 2017; Nie and Zhao, 2017; Ortizo et al., 2017; Pan et al., 2017; Wang et al., 2017a; Wang et al., 2017b; Zeng et al., 2017; Fu et al., 2018; Kuenzig et al., 2018; Shah et al., 2018; Han et al., 2019; Labarca et al., 2019; Piovani et al., 2019; Song et al., 2019; Yamada et al., 2019; Yang et al., 2019; Dai et al., 2020; Ji et al., 2020; Khorshidi et al., 2020; Lee et al., 2020; Li et al., 2020; Mozaffari et al., 2020; Phan et al., 2020; Rosillon et al., 2020; Agrawal et al., 2021; Bhagavathula et al., 2021; Khademi et al., 2021; Lorenzo-Pouso et al., 2021; Milajerdi et al., 2021; Piovani et al., 2021; Shirzad-Aski et al., 2021; Tian et al., 2021; Z et al., 2021; Zhang and He, 2021; B et al., 2022; D’Sa et al., 2022; Fatahi et al., 2022; Kermansaravi et al., 2022; Kim et al., 2022; Meisinger and Freuer, 2022; Milajerdi et al., 2022; Salavatizadeh et al., 2022; Shastri et al., 2022; Wang et al., 2022; Zhang et al., 2022; Zhao et al., 2022; Zhou et al., 2022; Zhang et al., 2023). In 2019, Piovani et al. (2019) published an umbrella review to assess environmental risk factors for IBD. They finally analyzed 71 environmental risk factors associated with IBD. However, they mainly focused on the external environmental factors and ignored the influence of internal environmental factors on IBD, such as obesity, depression and psoriasis. To our knowledge, there has been no comprehensive review and evidence assessment of all internal and external environmental risk factors associated with IBD. Before developing effective prevention strategies for IBD, it is necessary to systematically evaluate the quality of the methodology, potential biases, and validity of all studies available on the risk factors for IBD. Therefore, we conducted an umbrella review of meta-analyses to provide an overview of the evidence on IBD risk factors.

## 2 Methods and analysis

### 2.1 Design and registration

We systematically searched, extract, and analyze the data from reported systematic reviews and meta-analyses which focus on the risk factors of IBD following the Preferred Reporting Items for Systematic Reviews and Meta-Analysis (PRISMA) guidelines (Shamseer et al., 2015). The present umbrella review adhered to the methodological guidance outlined in the Joanna Briggs Institute Manual for Evidence Synthesis of Umbrella Reviews (Aromataris et al., 2022), along with following the procedures delineated in the Cochrane Handbook for conducting systematic reviews (Higgins et al., 2019).Furthermore, we have proactively enrolled our umbrella review in the International Prospective Register of Systematic Reviews (PROSPERO), with the registration number CRD42023417175. (https://www.crd.york.ac.uk/PROSPERO/).

### 2.2 Eligibility criteria

Systematic reviews and meta-analyses of non-interventional studies that evaluate the risk factors for IBD of any ethnicity or sex in all countries and settings are eligible for inclusion. Data on individual risk factors was extracted separately if two or more risk factors are reported in a single meta-analysis. If two or more meta-analyses (these studies are published more than 24 months apart) are performed on the same risk factor, we included the latest meta-analysis for data analysis. In the event that multiple meta-analyses are conducted within a 24-month timeframe, preference was given to the meta-analysis encompassing the highest number of prospective cohorts. If an equal number of prospective cohorts exists, priority was assigned to the meta-analysis with a superior AMSTAR score (Poole et al., 2017; Huang et al., 2023). Besides, if the latest meta-analysis does not perform dose-response analysis, while another meta-analysis does, both studies were included for data extraction. We excluded meta-analyses that evaluate the therapeutic effects of a certain treatment on IBD. Non-English studies, animal and cell culture studies also were excluded.

### 2.3 Population

This umbrella review is centered on systematically reviewing meta-analyses that assess the risk factors associated with IBD. The primary focus of the original articles incorporated within these systematic reviews and meta-analyses should be directed towards identifying factors that have the potential to either elevate or mitigate the risk of IBD. Studies evaluating the efficacy of a certain treatment for IBD, studies on the pathogenesis of IBD, and studies on the factors of IBD exacerbation and recurrence were excluded.

### 2.4 Exposure

We included meta-analysis which report at least 1 risk factors of IBD, including environmental, lifestyle, disease-related, treatment-related, demographic, genetic, social, and psychophysiological risk factors, etc. The strength of risk factors should be evaluated by odds ratio (OR), relative risk (RR) or hazard ratio (HR) with 95% confidence intervals (CIs).

### 2.5 Outcomes

The diagnosis of IBD in the original research should refer to the internationally recognized IBD diagnostic guidelines, such as ECCO-ESGAR Guideline for Diagnostic Assessment in IBD (Maaser et al., 2019), etc.

### 2.6 Study designs

Only systematic reviews and meta-analyses of non-interventional studies that evaluate the risk factors for IBD of any ethnicity or sex in all countries and settings are eligible for inclusion. All included systematic reviews and meta-analysis need to focus on the risk factors of IBD, and describe the meta-analysis method in detail, including complete search strategy, inclusion and exclusion criteria, literature quality evaluation criteria, result evaluation, analysis methods and procedures, and results interpretation criteria. The original performances included in systematic reviews included prospective or retrospective cohort designs, case-control studies, or cross-sectional studies.

### 2.7 Information sources

In our study, we systematically searched PubMed, Embase, Web of Science, and the Cochrane Database of Systematic Reviews until April 2023 for relevant systematic reviews and meta-analyses of non-interventional studies. We also reviewed the reference lists of included meta-analyses to find additional relevant articles.

### 2.8 Search strategy

The databases were accessed using Medical Subject Headings (MeSH), keywords, and text terms related to IBD, following the Scottish Intercollegiate Guidelines Network (SIGN) recommendations for literature search methodology: (((risk) OR (incidence)) AND ((systematic review) OR (meta-analysis))) AND (((inflammatory bowel diseases) OR (Inflammatory Bowel Disease)) OR (Bowel Diseases, Inflammatory)) (SIGN, 2020).

### 2.9 Study selection

**Figure 1:**
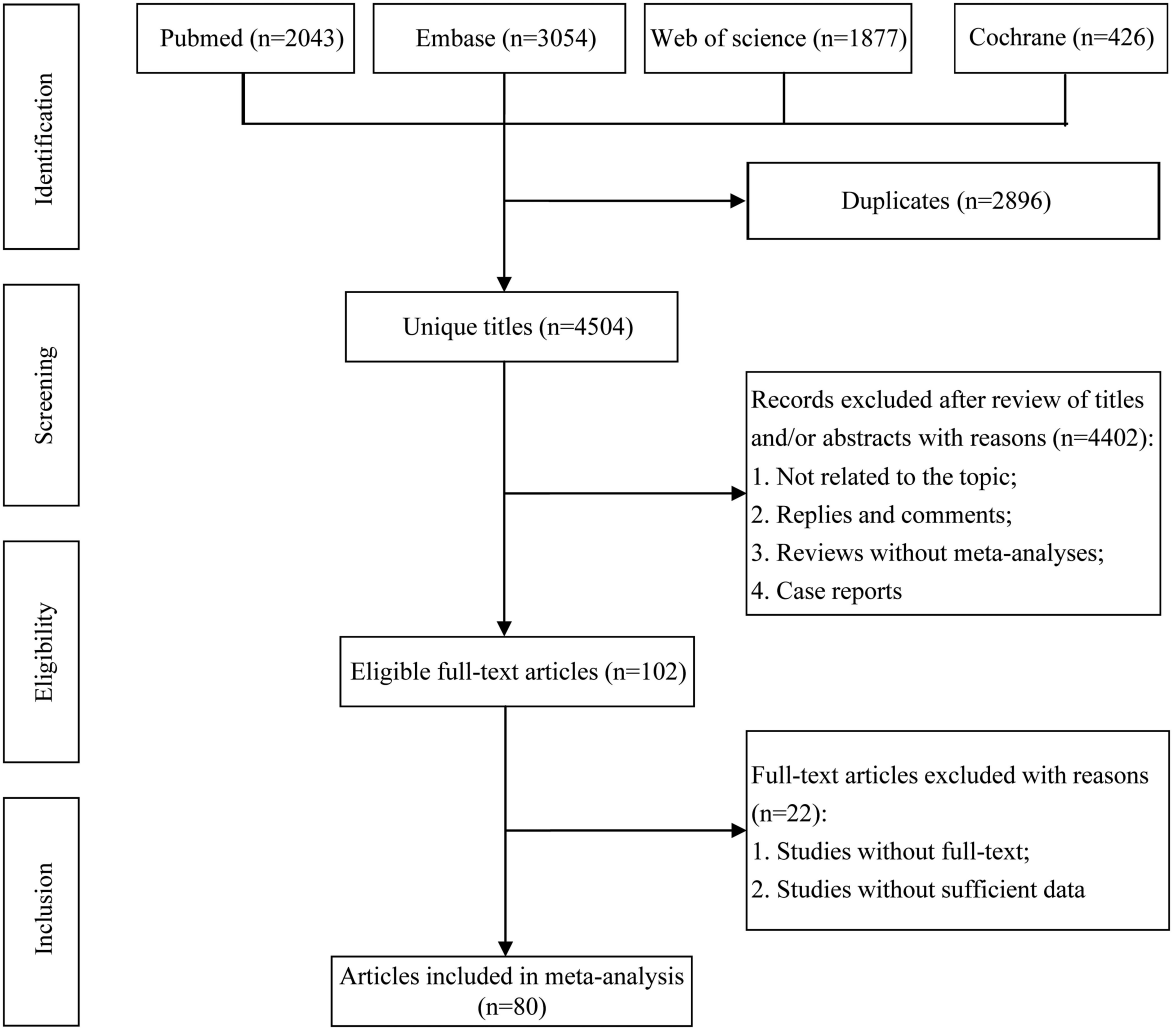
Flowchart of the systematic search and selection process.

All retrieved literature was screened using Endnote X9. After excluding duplicates, two authors screened the titles and abstracts and identify meta-analyses which meet the inclusion standard through full text reading independently. All disagreement in the process between the two authors were resolved by a third author. In addition, we hand searched studies from the reference lists to identify meta-analysis that might have been ignored (**Figure 1**).

### 2.10 Assessment of methodological quality

The methodological quality of each meta-analysis was assessed by two authors using AMSTAR, a validated, stringent, and reliable tool for evaluating systematic reviews and meta-analyses (Shea et al., 2007; Poole et al., 2017). Epidemiologic evidence for each risk factor was categorized into four classes using classification criteria: Class I (convincing evidence), Class II (highly suggestive evidence), Class III (suggestive evidence), Class IV (weak evidence), and NS (nonsignificant) (**Table 1**) (Ioannidis, 2009; Veronese et al., 2018; Wallace et al., 2020).

**Table 1:** Evidence classification criteria.

Evidence class	Description
Class I: convincing evidence	>1000 cases (or >20,000 participants for continuous outcomes), statistical significance at *P* < 10^−6^ (random-effects), no evidence of small-study effects and excess significance bias; 95% prediction interval excluded the null, no large heterogeneity (I^2^ < 50%)
Class II: highly suggestive evidence	>1000 cases (or >20,000 participants for continuous outcomes), statistical significance at *P* < 10^−6^ (random-effects) and largest study with 95% CI excluding the null value
Class III: suggestive evidence	>1000 cases (or >20,000 participants for continuous outcomes) and statistical significance at *P* < 0.001
Class IV: weak evidence	The remaining significant associations with *P* < 0.05
NS: non-significant	*P* > 0.05

### 2.11 Data extraction

Two investigators autonomously retrieved the pertinent data from each qualifying study: 1) name of author, 2) publication time, 3) risk factors, 4) type of IBD (all IBD, CD, or UC), 5) number of included studies, 6) number of cases and total participants, 7) study design (cross-sectional, case-control, cohort), 8) length of follow-up, and 9) RR, OR, or HR estimates with 95% CIs. In addition, we extracted the meta-analytic model used (random or fixed), estimate of heterogeneity (*I* ^2^ and Cochran’s *Q*-test) and small-study assessment (Egger’s test, Begg’s test and funnel plot). When dose-response analysis and subgroup analysis are performed, we extracted the *P* value for nonlinearity and any reported estimate for subgroup analysis. Any disagreement was resolved by a third author.

### 2.12 Data summary

We recalculated the RR, OR, or HR with 95% CIs through random or fixed effects models and evaluate the heterogeneity (*I* ^2^ and Cochran’s *Q*-test) and small-study effects (Egger or Begg test for each systematic review and meta-analysis with over 10 studies) in each meta-analysis when sufficient data are provided (Egger et al., 1997; Theodoratou et al., 2014; Huang et al., 2021). Risk factors included the following five aspects according to the health ecological model (Kennedy et al., 2021; Lu et al., 2021): innate personal trait (including age, gender, race, genetics, birth status, height, weight, BMI, underlying diseases, previous treatments, etc.), behavioral lifestyles (including diet, exercise, smoking, drinking, staying up late, working hours, etc.), interpersonal network (including marriage, family relationship, social relationship, etc.), socioeconomic status (including occupation, family economic level, debt, etc.), macro-environments (including urban or rural environment, pets, immigrants, residential environment, etc.).

For risk factors identified as class I or II evidence, we conducted sensitivity analysis when sufficient data are provided to identify the effect of some individual study on total significance of the evidence. Dose-response analysis for any risk factors of IBD was also extracted from the involved meta-analyses. Furthermore, if the most recent meta-analysis does not involve the clinical researches which are involved by other meta-analyses, we combined the data of these studies and perform a re-analysis.

A *P* < 0.10 is considered significant for heterogeneity tests, and for other tests, *P* < 0.05 is deemed significant. Review Manager v5.4 (Cochrane Collaboration, Oxford, UK) is used for evidence synthesis. Egger and Begg tests, along with sensitivity analysis, are performed using Stata v15.1.

## 3 Major outcomes

### 3.1 Characteristics of meta-analyses

The flowchart of the literature search and selection process are presented in **Figure 1**. After a systematic literature search, 4,504 unique articles were identified. A total of 80 meta-analyses were yielded based on our inclusion criteria. We extracted 191 unique risk factors in meta-analyses, including 92 significantly associated risk factors (**Table 2**) and 99 non-significantly associated risk factors. The full version of the risk factors if IBD are presented in **Supplementary Table S1**. A total of 62 adverse associations and 30 favorable associations showed statistical significance in our analysis. After a careful evaluation of evidence quality using established criteria, most observed outcomes were classified as IV (low quality) or NS (non-significant) evidence. Notably, only 10 risk factors (5.2%) achieved class II and III evidence in this comprehensive review. Importantly, underlying medical conditions were the main contributors to the risk of IBD.

**Table 2:** Significantly-associated risk factors for IBD.

Risk factors	Exposure contrast	Diseases	Total eligible MA	Included MA	No. of cases/total	MA metric	Estimates, 95% CI	No. of studies T/R/C/P	Effects model	I^2^; Q test P value	Egger test P value	AMSTAR	Evidence classification
Antibiotic use	Any vs. none	CDwest	3	Zhao2021	NA/6,024^a,b^	OR	1.90, 1.60-2.27	18/0/5/13	Random	84.1;0	0.148	8	IV
Antibiotic use	Any vs. none	UCwest	3	Zhao2021	NA/4,996^a,b^	OR	1.40, 1.12-1.76	15/0/5/10	Random	86;0	0.884	8	IV
Appendectomy	Yes vs. no	CD	3	Zhang2023	2,751/2,240,604^a^	RR	2.28, 1.66-3.14	6/0/6/0	Random	97.4;0.000	0.548	9	II
Asthma	Yes vs. no	CD	1	Kuenzig2018	18,083/NA^a,b^	RR	1.31, 1.16-1.47	15/0/3/12	Random	88;<0.01	NA	9	II
Asthma	Yes vs. no	UC	1	Kuenzig2018	18,329/NA^a,b^	RR	1.30, 1.21-1.40	16/0/3/13	Random	93;<0.01	NA	9	II
Atopic dermatitis	Yes vs. no	UC	2	Lee 2020	266/74,380^a^	OR	1.54, 1.07-2.18	3/0/3/0	Random	65; 0.06	NA	8	IV
Autism spectrum disorder	Yes vs. no	IBD	1	Kim2022	23,506/115,297^a,b^	OR	1.57, 1.28-1.930	6/0/1/5	Random	87.1;<0.001	NA	9	II
Bariatric Surgery	Before vs. after	IBD	1	Kermansaravi2022	708/149,385^a^	OR	1.17, 1.06-1.29	4/0/4/0	Random	90.2;0.000	0.677	9	IV
BMI	Highest vs. lowest	IBD	2	Milajerdi2022	NA/1,503,262^a^	OR	0.76, 0.66-0.88	5/0/5/0	Random	93.2;0.000	NA	8	III
Breast feeding	Yes vs. no	CDwest	4	Zhao2021	NA/7,011^a,b^	OR	0.87, 0.76-1.00	24/0/4/0	Random	74.9;0	0.041	8	IV
Breast feeding	Yes vs. no	CDeast	4	Zhao2021	NA/228^a,b^	OR	0.29, 0.11-0.81	2/0/0/2	Random	78.9;0.007	0.041	8	IV
Campylobacter species	Yes vs. no	IBD	2	Castaño-Rodríguez2017	519/NA^b^	OR	2.969, 1.330-6.626	9/0/0/9	Random	82.03;0.008	0.104	7	IV
Ceasarean	Yes vs. no	CDwest	3	Zhao2021	NA/4892^a,b^	OR	1.16, 1.05-1.28	9/0/1/8	Random	18.8;0.270	NA	8	IV
Ceasarean	Yes vs. no	UCwest	3	Zhao2021	NA/2131^a,b^	OR	1.17, 1.08-1.27	7/0/1/6	Random	0; 0.493	NA	8	IV
Cholesterol	Highest vs. lowest	UCeast	1	Zhao2021	NA/307^b^	OR	1.66, 1.26-2.20	4/0/0/4	Random	0;0.767	NA	8	IV
Chronic obstructive pulmonary diseases	Yes vs. no	CD	1	Labarca2019	NA/675^a^	RR	2.29, 1.51-3.48	4/0/4/0	Random	62;0.05	NA	10	IV
Chronic obstructive pulmonary diseases	Yes vs. no	UC	1	Labarca2019	NA/680^a^	RR	1.79, 1.39-2.29	4/0/4/0	Random	19;0.30	NA	10	IV
Chronic obstructive pulmonary diseases	Yes vs. no	CD/UC	1	Labarca2019	NA/1355^a^	RR	2.02, 1.56-2.63	4/0/4/0	Random	72;0.0008	NA	10	IV
Coffee	Highest vs. lowest	UCwest	3	Zhao2021	NA/1,072^b^	OR	0.58, 0.40-0.83	6/0/0/6	Random	75.9;0	NA	8	IV
Coffee	Highest vs. lowest	UCeast	3	Zhao2021	NA/306^b^	OR	0.53, 0.39-0.73	3/0/0/3	Random	0;0.501	NA	8	IV
Contact with farm animals	Yes vs. no	CDwest	2	Zhao2021	NA/1137^b^	OR	0.70, 0.54-0.91	4/0/0/4	Random	68.2;0.001	NA	8	IV
Dietary fat	High vs. low	IBD,UC,CD	2	Wu2016	1,084/398,081^a,b^	ES	1.52, 1.16-1.99	8/0/3/5	Random	0;0.527	0.728	8	IV
Dietary fiber intake	Highest vs. lowest	IBD	4	Milajerdi2021	NA/2,758^a,b^	RR	0.83, 0.70-0.97	6/0/3/3	fixed	45.6;0.037	NA	8	IV
Dietary fiber intake	Highest vs. lowest	CD	4	Milaherdi2021	NA/2,518^a,b^	ES	0.59, 0.46-0.74	5/0/3/2	fixed	0;0.869	NA	8	IV
Dietary fiber intake	Highest vs. lowest	CD	4	Milaherdi2021	NA/2,518^a,b^	ES	0.59, 0.46-0.74	5/0/3/2	fixed	0;0.869	NA	8	IV
Egg	Highest vs. lowest	UCeast	1	Zhao2021	NA/613^b^	OR	1.37, 1.12-1.68	6/0/0/6	Random	0.8;0.436	0.189	8	IV
Exposure to pets	Yes vs. no	CDeast	2	Zhao2021	NA/460^a^	OR	0.54, 0.38-0.77	3/0/0/3	Random	68.0;0.002	0.42	8	IV
Exposure to pets	Yes vs. no	UCwest	2	Zhao2021	NA/1966^b^	OR	0.73, 0.60-0.88	11/0/0/11	Random	62.8;0	0.277	8	IV
Exposure to pets	Yes vs. no	UCeast	2	Zhao2021	NA/848^b^	OR	0.66, 0.47-0.93	3/0/0/3	Random	72.9;0	0.277	8	IV
Exposure to pets	Yes vs. no	CDwest	2	Zhao2021	NA/529^b^	OR	0.54, 0.38-0.77	3/0/0/3	Random	22.7;0.248	NA	8	IV
Fatty acids	Highest vs. lowest	UCeast	1	Zhao2021	NA/253^b^	OR	1.43,1.19-1.72	3/0/0/3	Random	16.3;0.226	0.02	8	IV
Fiber intake	Per 10 g increment/day	CD	3	Zeng2017	NA/580^a,b^	RR	0.853, 0.762-0.955	4/0/1/3	Random	0;0.730	0.708	8	IV
Fruit	Highest vs. lowest	UC	5	Milajerdi2021	NA/2,154^a^	RR	0.69, 0.55-0.86	4/0/4/0	fixed	87.0;0.000	NA	8	IV
Fruit	Highest vs. lowest	CD	5	Milajerdi2021	NA/2154^a^	RR	0.47,0.38-0.58	4/0/4/0	fixed	32.1;0.220	NA	8	IV
Fruit	Highest vs. lowest	IBD	5	Milajerdi2021	NA/2154^a^	RR	0.56, 0.48-0.65	4/0/4/0	fixed	79.0;0.000	NA	8	IV
HCMV infection	Yes vs. no	I BD	1	Lv2017	135/529^b^	OR	4.99, 2.40-10.40	12/0/0/12	fixed	66;<0.0001	NA	8	IV
Healthy/prudent dietary patterns	Yes vs. no	CD	1	Khorshidi2020	NA/NA^a,b^	OR	0.39, 0.16-0.62	5/0/2/3	Random	67.9;0.014	0.142	8	IV
Helicobacter pylori	Yes vs. no	IBD	8	Bouriat2022	2,545/NA^b^	OR	0.42, 0.32-0.56	19/0/0/19	Random	NA;<0.0001	NA	8	II
Hidradenitis suppurativa	Yes vs. no	CD	2	Phan2020	NA/101,940^b^	OR	2.25, 1.52-3.32	5/0/0/5	Random	92;<0.00001	NA	10	III
Hidradenitis suppurativa	Yes vs. no	UC	2	Phan2020	NA/39,455^b^	OR	1.56, 1.26-1.94	3/0/0/3	Random	36;0.21	NA	10	II
Hidradenitis suppurativa	Yes vs. no	Unspecified IBD	2	Phan2020	NA/9,221^b^	OR	2.80, 1.81-4.35	2/0/0/2	Random	67;0.08	NA	10	IV
IBD family history	Yes vs. no	UCwest	1	Zhao2021	NA/3,880^b^	OR	3.64, 2.85-4.63	18/0/1/17	Random	52.5;0.003	0	8	IV
IBD family history	Yes vs. no	UCeast	1	Zhao2021	NA/1,281^b^	OR	3.88, 2.54-5.94	6/0/0/6	Random	0;0425	0	8	IV
IBD family history	Yes vs. no	CDwest	1	Zhao2021	NA/6,369^b^	OR	4.09, 3.49-4.81	25/0/1/24	Random	36.1;0.029	0	8	IV
IBD family history	Yes vs. no	CDeast	1	Zhao2021	NA/523^b^	OR	4.39, 2.55-7.59	4/0/0/4	Random	0;0.726	0	8	IV
Isotretinoin	Yes vs. no	UCwest	2	Zhao2021	NA/14,214^a,b^	OR	1.48, 1.06-2.07	5/0/1/4	Random	41.8;0.127	NA	8	IV
Meat and meat product	Highest vs. lowest	CDwest	3	Zhao2021	NA/1,419^a,b^	OR	1.58, 1.20-2.08	2007/1/6	Random	83.5;0	NA	8	IV
Milk and dairy products	Highest vs. lowest	CDwest	1	Zhao2021	NA/1077^a,b^	OR	0.79, 0.65-0.97	8/0/1/7	Random	64.6;0	0.281	8	IV
More physical activity	Yes vs. no	CDwest	1	Zhao2021	NA/1,055^a,b^	OR	0.67, 0.55-0.83	8/0/2/6	Random	54.5;0.004	0.028	8	IV
More physical activity	Yes vs. no	CDeast	1	Zhao2021	NA/1,625^a,b^	OR	0.73, 0.58-0.93	3/0/1/2	Random	62.9;0.019	0.028	8	IV
More physical activity	Yes vs. no	UCeast	1	Zhao2021	NA/934^b^	OR	0.63, 0.51-0.78	2/0/0/2	Random	21.5;0.281	0.289	8	IV
MUFA	Highest vs. lowest	UCeast	3	Zhao2021	NA/253^b^	OR	1.66, 1.19-2.32	3/0/1/2	Random	22.6;0.242	NA	8	IV
Multiple birth	Yes vs. no	CDwest	2	Zhao2021	NA/14,758^a,b^	OR	0.76, 0.60-0.97	10/0/1/9	Random	68.6;0	0.619	8	IV
Multiple birth	Yes vs. no	UCeast	2	Zhao2021	NA/1,273^b^	OR	0.79, 0.66-0.95	2/0/0/2	Random	0;0.393	0.625	8	IV
Multiple sclerosis	Yes vs. no	UC	1	Wang2022	127/1,280^b^	RR	1.42, 1.17-1.71	NA	Fixed	38;0.0003	0.196	8	IV
Multiple sclerosis	Yes vs. no	CD	1	Wang2022	107/977^b^	RR	1.41, 1.14-1.74	NA	Fixed	0;0.001	0.196	8	IV
Multiple sclerosis	Yes vs. no	UC and CD	1	Wang2022	234/2,267^b^	RR	1.41, 1.23-1.63	NA	Fixed	0;<0.00001	0.196	8	IV
n-6PUFA	Highest vs. lowest	UCwest	1	Zhao2021	NA/533^a,b^	OR	1.25, 1.01-1.54	4/0/0/1	Random	32.5;0.139	NA	8	IV
Number of siblings	High vs. low	CD	1	Cholapranee2016	NA/13,185^b^	OR	0.93, 0.88-0.98	5/0/0/5	Random	NR	NR	8	IV
Oral contraceptive	Yes vs. no	CDwest	4	Zhao2021	NA/4,852^a,b^	OR	1.31, 1.12-1.53	24/0/2/22	Random	54.2;0	0.034	8	IV
Oral contraceptive	Yes vs. no	UCweat	4	Zhao2021	NA/4,045^a,b^	OR	1.18, 1.03-1.35	19/0/1/18	Random	0;0.877	0.97	8	IV
Otitis media infection	Yes vs. no	IBD	1	Agrawal2021	NA/17967^a^	OR	2.11, 1.22-3.62	2/0/2/0	Random	36.9;0.21	NA	9	IV
Otitis media infection	Yes vs. no	CD	1	Agrawal2021	NA/10,318^a^	OR	1.95, 1.20-3.17	2/0/2/0	Random	4.41;0.31	NA	9	IV
Periodontal	Yes vs. no	IBD	2	Lorenzo-Pouso2021	657/NA^a,b^	RR	2.78, 1.36-5.69	8/0/1/7	Random	87.89;0.00001	0.008	9	IV
Periodontal	Yes vs. no	CD	2	Lorenzo-Pouso2021	619/NA^a,b^	RR	3.41, 1.36-8.56	7/0/1/6	Random	946;0.0001	0.008	9	IV
Periodontal	Yes vs. no	UC	2	Lorenzo-Pouso2021	409/NA^a,b^	RR	3.98, 2.02-7.87	7/0/1/6	Random	72.88;000001	0.008	9	IV
Personal toilet	Yes vs. no	UC	1	Cholapranee2016	NA/20,365^a^	OR	0.73, 0.59-0.88	9/0/1/8	Random	NA	<0.05	8	III
Poliomyelitis	Yes vs. no	CD	1	Pineton2015	NA/345^b^	RR	2.28, 1.12-4.68	2/0/0/2	Random	0;0.606	NA	8	IV
Poliomyelitis	Yes vs. no	UC	1	Pineton2015	NA/174^b^	RR	3.48, 1.24-9.71	2/0/0/2	Random	0;0.5397	NA	8	IV
Proton pump inhibitors, PPIs	Any vs. none	IBD	1	Shastri2022	NA/12,714^a,b^	OR	2.43, 1.18-5.02	6/0/1/5	Random	86;0.00001	NA	9	IV
Psoriasis	Yes vs. no	CD	1	Fu2018	NA/85,761^a^	RR	2.53, 1.65-3.89	4/0/4/0	Random	55;0.08	NA	8	IV
Psoriasis	Yes vs. no	UC	1	Fu2018	NA/85,761^a^	RR	1.71, 1.55-1.89	4/0/4/0	Random	0;0.55	NA	8	IV
Rosacea	Yes vs. no	IBD	2	Han2019	NA^c^	ES	1.32, 1.18-1.49	NA	Random	31.6;0.187	>0.05	8	IV
Smoking	Any vs. no	CDwest	4	Zhao2021	NA/10021^a,b^	OR	1.61, 1.47-1.77	53/0/4/49	Random	64.4;0	0.017	8	II
Smoking	Any vs. no	CDeast	4	Zhao2021	NA/1550^a,b^	OR	1.29, 1.09-1.52	11/0/1/10	Random	49.2;0.008	0.017	8	IV
Soft drink	Highest vs. lowest	CDwest	3	Zhao2021	NA/1,735^a,b^	OR	1.56, 1.25-1.95	8/0/1/7	Random	56.7;0.002	0.965	8	IV
Suger	Highest vs. lowest	UC	2	Khademi2021	NA/1,213^a,b^	RR	1.59, 1.15-2.20	4/0/2/2	Random	0;0.58	NA	8	IV
Suger	Highest vs. lowest	CD	2	Khademi2021	NA/1,213^a,b^	RR	1.90, 1.06-3.14	4/0/2/2	Random	57.5;0.051	NA	8	IV
Suger	Highest vs. lowest	IBD	2	Khademi2021	NA/1,213^a,b^	RR	1.71, 1.24-2.38	4/0/2/2	Random	41.5;0.14	NA	8	IV
Tea consumption	Highest vs. lowest	CD	3	Yang2019	NA/288^b^	RR	0.70, 0.53-0.93	2/0/0/2	Random	0;0.636	NA	8	IV
Tea consumption	Highest vs. lowest	UC	3	Nie2017	NA/1,741^b^	RR	0.69, 0.58-0.83	3/0/0/3	Random	0;0.697	0.623	8	IV
Tonsillectomy	Yes vs. no	CDwest	2	Zhao2021	NA/28,971^a,b^	OR	1.27, 1.09-1.46	14/0/1/13	Random	66.8;0	0.39	8	IV
Total energy	Highest vs. lowest	CDwest	1	Zhao2021	NA/227^b^	OR	1.44, 1.10-1.90	2/0/0/2	Random	0;0.703	NA	8	IV
Urban living	Yes vs. no	CDwest	4	Zhao2021	NA/3,360^b^	OR	1.42, 1.28-1.56	27/0/0/27	Random	86.2;0	0.071	8	IV
Urban living	Yes vs. no	UCwest	4	Zhao2021	NA/2,436^b^	OR	1.22,1.11-1.34	4/0/0/4	Random	0;0.773	0.26	8	IV
Vegetables	Highest vs. lowest	CDwest	5	Zhao2021	NA/1,033^a,b^	OR	0.67, 0.57-0.79	10/0/3/7	Random	43.2;0019	0.041	8	IV
Vitamin D	Highest vs. lowest	CDwest	5	Zhao2021	NA/684^b^	OR	1.58, 1.17-2.13	13/0/0/13	Random	27.3;0.169	0.029	8	IV
Vitamin D	Highest vs. lowest	UCwest	5	Zhao2021	NA/380^b^	OR	1.98, 1.17-3.37	8/0/0/8	Random	59.8;0.015	0.252	8	IV
Vitamin D	Highest vs. lowest	UCeast	5	Zhao2021	NA/121^b^	OR	2.20, 1.30-3.72	3/0/0/3	Random	0;0.414	0.252	8	IV
Western dietary pattern	Yes vs. no	CD	1	Li2020	NA/707^a,b^	RR	1.72, 1.01-2.93	7/0/3/4	Random	74.8;0.000	NA	8	IV
Western dietary pattern	Yes vs. no	UC	1	Li2020	NA/683^a,b^	RR	2.15, 1.38-3.34	8/0/3/5	Random	65.9;0.005	NA	8	IV
Western dietary pattern	Yes vs. no	IBD, CD and UC	1	Li2020	NA/1390^a,b^	RR	1.92, 1.37-2.68	8/0/3/5	Random	70.4;0.000	NA	8	IV

CD, Crohn’s disease; UC, ulcerative colitis; IBD, Inflammatory bowel disease; BMI, Body Mass Index; HCMV, human cytomegalovirus; MUFA, monounsaturated fatty acid; PUFA, polyunstatured fatty acid; BGG, Bacillus Calmette Guerin; EHS, Enterohepatic Helicobacter Species; NA, Not Available; OR, Odds ratio; RR, Relative risk; HR, Hazard ratio; ES, Effect Sizes; pOR, pooled OR.

a. Cohort.

b. Case-control.

c. Unclear.

### 3.2 Class II and III evidence

**Figure 2:**
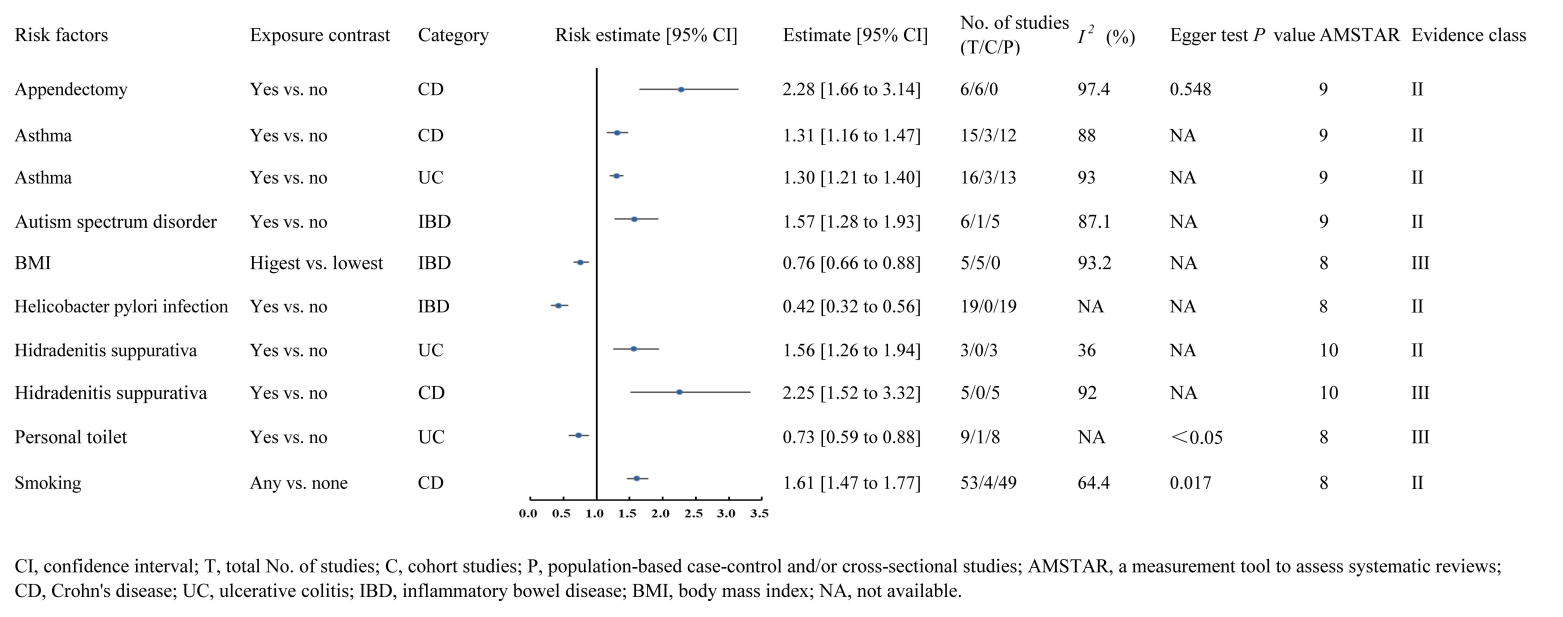
Forest plots of IBD risk factors for class II and class III evidence.

A meta-analysis of 6 cohorts including 2,240,604 participants and 2,751 cases found that appendectomy was significantly associated with an increased risk of CD (RR 2.28, 95% CI 1.66-3.14) (class II evidence) (Zhang et al., 2023). In addition, patients with asthma were observed with an increased risk of CD compared those without asthma (RR 1.31, 95% CI 1.16-1.47) (class II evidence) (Kuenzig et al., 2018). Meanwhile, patients with asthma were also observed with an increased risk of UC compared those without asthma (RR 1.30, 95% CI 1.21-1.40) (class II evidence) (Kuenzig et al., 2018). Furthermore, another meta-analysis of 1 cohort and 5 case-control studies including 115,297 participants and 23,506 cases found that autism spectrum disorder was significantly associated with an increased risk of IBD (OR 1.57, 95% CI 1.28-1.93) (class II evidence) (Kim et al., 2022). On the other hand, a meta-analysis of 5 cohorts including 1,503,262 participants found that compared with populations with lower BMI, people with higher BMI had a significantly decreased risk of IBD (OR 0.76, 95% CI 0.66-0.88) (class III evidence) (Milajerdi et al., 2022). Besides, another meta-analysis of 19 case-controls including 2,545 cases found that helicobacter pylori infection was significantly associated with a decreased risk of IBD (OR 0.42, 95% CI 0.32-0.56) (class II evidence) (B et al., 2022). Evidence from a meta-analysis of 5 case-control studies found that patients with hidradenitis suppurativa were observed with an increased risk of CD compared those without hidradenitis suppurativa (OR 2.25, 95% CI 1.52-3.32) (class III evidence) (Phan et al., 2020). In addition, this meta-analysis also found that patients with hidradenitis suppurativa were also observed with an increased risk of UC compared those without hidradenitis suppurativa (OR 1.56, 95% CI 1.26-1.94) (class II evidence) (Phan et al., 2020). With respect of living condition, a meta-analysis of 1 cohort and 8 case-control studies including 20,365 participants found that people who had a personal toilet had a significantly lower risk of UC than those who did not (OR 0.73, 95% CI 0.59-0.88) (class III evidence) (Cholapranee and Ananthakrishnan, 2016). Finally, another large-scale meta-analysis including 4 cohorts and 49 case-control studies found that western populations with any history of smoking had a significantly higher risk of CD compared to non-smokers (OR 1.61, 95% CI 1.47-1.77) (class II evidence) (Zhao et al., 2022) (**Figure 2**).

### 3.3 Class IV and NS evidence

This umbrella review included a total of 82 risk factors with class IV evidence, of which 27 were protective factors and 55 were risk factors. For protective factors, the top ten effect values were breast feeding (OR 0.29, 95% CI 0.11-0.81) (Zhao et al., 2022), healthy/prudent dietary patterns (OR 0.39, 95% CI 0.16-0.62) (Khorshidi et al., 2020), fruit consumption (RR 0.47, 95% CI 0.38-0.58) (Milajerdi et al., 2021), coffee consumption (OR 0.53, 95% CI 0.39-0.73) (Zhao et al., 2022), exposure to pets (OR 0.54, 95% CI 0.38-0.77) (Zhao et al., 2022), dietary fiber intake (OR 0.59, 95% CI 0.46-0.74) (Milajerdi et al., 2021), more physical activity (OR 0.63, 95% CI 0.51-0.78) (Zhao et al., 2022), vegetables intake (OR 0.67, 95% CI 0.57-0.79) (Zhao et al., 2022), tea consumption (RR 0.69, 95% CI 0.58-0.83) (Nie and Zhao, 2017), and contact with farm animals (OR 0.70, 95% CI 0.54-0.91) (Zhao et al., 2022). For risk factors, the top ten effect values were human cytomegalovirus (HCMV) infection (OR 4.99, 95% CI 2.40-10.40) (Lv et al., 2017), IBD family history (OR 4.39, 95% CI 2.55-7.59) (Zhao et al., 2022), periodontal disease (RR 3.98, 95% CI 2.02-7.87) (Lorenzo-Pouso et al., 2021), poliomyelitis (OR 3.48, 95% CI 1.24-9.71) (Pineton de Chambrun et al., 2015), campylobacter species infection (OR 2.969, 95% CI 1.330-6.626) (Castaño-Rodríguez et al., 2017), hidradenitis suppurativa (OR 2.80, 95% CI 1.81-4.35) (Phan et al., 2020), psoriasis (RR 2.53, 95% CI 1.65-3.89) (Fu et al., 2018), use of proton pump inhibitors (OR 2.43, 95% CI 1.18-5.02) (Shastri et al., 2022), chronic obstructive pulmonary disease (RR 2.29, 95% CI 1.51-3.48) (Labarca et al., 2019), and western dietary pattern (RR 2.15, 95% CI 1.38-3.34) (Li et al., 2020). In addition, a total of 99 non-significant risk factors were reported in this umbrella review and detailed data of all risk factors are shown in **Supplementary Table S1**.

### 3.4 Heterogeneity

In our study, 53% of total risk factors were reanalyzed using a random or fixed effects model. The reanalysis revealed that about 50.3% of examined risk factors exhibited noteworthy heterogeneity (*I* ^2^ > 50% or Cochran’s Q-test *P* < 0.1). Most outcomes’ observed heterogeneity could be attributed to various potential factors, such as study setting, geographical region, ethnicity, gender, age, study quality, design, sample size, follow-up duration, and adjustment for confounding variables. For the remaining 47% of unanalyzed risk factors, approximately 60% showed significant heterogeneity, and 4.2% did not report results for the heterogeneity assessment.

### 3.5 Assessment of risk of bias

In our reanalysis, Egger’s test assessed publication bias for 48.7% of identified risk factors, revealing bias in 30 of them. For non-reanalyzed outcomes, publication bias was detected in 33% of risk factors via statistical tests or funnel plots. Importantly, other outcomes either showed no significant publication bias or lacked reported bias assessments.

### 3.6 AMSTAR score

The median AMSTAR score for all identified risk factors was 8 (7-10) as outlined in **Supplementary Table S1**. Further detailed AMSTAR scores specific to each outcome can be found in **Supplementary Table S2**.

## 4 Discussion

### 4.1 Principal findings and possible explanations

IBD occurs in working adults aged 20 to 40, and the prevalence is similar in men and women, which has a great negative impact on the quality of life and work of patients. At present, there is a lack of clear understanding of the specific etiology and pathogenesis of IBD, and at the same time, it has caused a heavy burden on the global health system due to its characteristics of recurrent symptoms, poor effect of drug treatment and surgical intervention. Up to now, a large number of researchers around the world have carried out clinical research and evidence-based medical research on the risk factors of IBD. The umbrella evaluation evaluated the advantages and disadvantages of existing evidence-based medical from systematic review and meta-analyses on the risk factors of IBD, helped to understand the potential risk factors for the occurrence and development of IBD in a more comprehensive way from multiple dimensions, provided a theoretical basis for the development of more clinical effective prevention and control measures for IBD, and provided directions for further clinical research.

The present umbrella review extracted 191 unique risk factors including 92 significantly associated risk factors and 99 non-significantly associated risk factors. Among these, statistical significance was attained for 62 adverse associations and 30 favorable associations. After a rigorous quality assessment of the evidence using established classification criteria, most observed outcomes were categorized as either Class IV or NS evidence.

Only 10 (5.2%) risk factors were graded as class II and III evidence in this umbrella review, including appendectomy, asthma (UC), asthma (CD), autism spectrum disorder, BMI, helicobacter pylori infection, hidradenitis suppurativa (UC), hidradenitis suppurativa (CD), personal toilet and smoking.

First, we found that appendectomy is a risk factor for CD (class II evidence), and several potential mechanisms support this study finding. The appendix contains abundant gut-associated lymphoid tissue (GALT) with helper T lymphocytes and B lymphocytes (plasma cells) generating diverse immunoglobulins. It notably shows a high concentration of immune cells producing immunoglobulin A (IgA) and immunoglobulin G (IgG). The synthesis of Ig is thought to be crucial in modulating the abundance and composition of the gut microbiota (Zahid, 2004; Girard-Madoux et al., 2018). Andreu Ballester et al. reported a significant decrease in serum IgA levels following appendectomy, persisting for at least three years (Andreu-Ballester et al., 2007). Additionally, the gastrointestinal microbiota plays a crucial role in CD pathogenesis (Wright et al., 2017). Some studies suggest that the biofilm in the appendix may act as a protective enclave, aiding the reintroduction of beneficial microorganisms after a gastrointestinal infection (Bollinger et al., 2007). Disrupting biofilm formation may induce dysbiosis, making tissues prone to inflammation and contributing to Crohn’s disease progression.

Second, we found that a large number of immune diseases are risk factors for IBD, with asthma being a risk factor for UC and CD (class II evidence). IBD and asthma are immune-mediated disorders with shared genetic and environmental risk factors. Barrier function anomalies in the lung and gastrointestinal epithelium, along with abnormal immune responses to environmental stimuli and pathogens, are common features in both conditions (de Souza and Fiocchi, 2016). The “hygiene hypothesis” applies to IBD and asthma, suggesting that individuals raised in sterile environments during childhood are more prone to developing chronic immune-mediated diseases later in life. Early-life disturbances in gut microbiota may increase disease susceptibility. For example, early antibiotic exposure raises asthma and IBD prevalence, while breastfeeding protects against both conditions (Dogaru et al., 2014). Inadequate early-life exposure to gut pathogens may increase vulnerability to immune-mediated diseases, including asthma and IBD (Stiemsma et al., 2015). In addition, we found that autism spectrum disorder is a risk factor for IBD (class II evidence). A number of pathophysiological hypotheses focusing on putative gut-brain connectivity have been proposed to explain the possible link between autism spectrum disorder and IBD, including dysfunction of the intestinal tight junction in autism spectrum disorder, leading to altered intestinal permeability (D’Eufemia et al., 1996), and, in comparison to neuronormal children, the risk of intestinal permeability changes. Children with autism spectrum disorder (ASD) display variations in their gut microbiota (Adams et al., 2011; Gondalia et al., 2012). A suggested shared genetic basis between ASD and IBD exists (Solmi et al., 2020). Several observational studies have documented a statistically significant correlation between ASD and IBD (Doshi-Velez et al., 2015; Alexeeff et al., 2017; Lee et al., 2018; Butwicka et al., 2019).

Interestingly, this study found that *H. pylori* infection was a protective factor for IBD (class II evidence), a conclusion not supported by a large number of prospective clinical studies. The protective effect is attributed to the generation of interleukin-18 (IL-18) by regulatory T cells (Luther et al., 2011). An alternative immunoregulatory mechanism is also suggested, involving the secretion of Helicobacter pylori neutrophil-activating protein. This protein reduces inflammation by stimulating toll-like receptor 2 through agonist ligation. Additionally, Helicobacter pylori DNA has been identified as another factor in this regulatory process, showing a preventive impact on sodium dextran sulfate-induced colitis in murine models (Michetti, 2004). Furthermore, this study found that hidradenitis suppurativa is a risk factor for UC (class II evidence) and CD (class III evidence). Hidradenitis suppurativa and IBD are both thought to have inflammatory components with similar histological manifestations (Roy et al., 1997). Both diseases have been shown to respond to TNF-α antibodies (including infliximab and Adalizumab) involving inflammatory pathways involving IL-23 and Th-17 that have been linked to hidradenitis suppurativa and Crohn’s disease (Abraham and Cho, 2009; Schlapbach et al., 2011). Both the skin and the intestine are densely vascularized, innervated organs that share structural and functional commonalities. Both of these organs are the main interface to the external environment and therefore play a key role in homeostasis. There is evidence of a bidirectional link between the gut and the skin, so the proposed skin-gut axis supporting this is the skin manifestation of several gastrointestinal disorders, including cutaneous Crohn’s disease (O’Neill et al., 2016). The gut microbiome has also been shown to be involved in skin diseases including acne, atopic dermatitis and psoriasis. Changes in the microbiome in Crohn’s disease and ulcerative colitis may have a modulating effect on systemic immunity (Kostic et al., 2014). Evidence from this study also suggests that the gut microbiome may have an impact on the skin microbiome, highlighting a new and least explored area in hidradenitis suppurativa (Schwarz et al., 2017).

On the other hand, we found that patients with high BMI had a lower risk of developing IBD (class III evidence). The precise mechanism of this protective relationship is unclear. However, differences in gut microbiota between obese and normal-weight patients may explain this finding. Overweight and obesity are associated with changes in the gut microbiome that may increase the production of short-chain fatty acids (SCFAs) (Schwiertz et al., 2010). The main bacterial groups in fecal samples of individuals with obesity were Firmicutes and Bacteroidetes. The Firmicutes to Bacteroidetes ratio showed variations in both overweight and obese subjects (Schwiertz et al., 2010). Changes in this ratio, along with increased short-chain fatty acid (SCFA) production, indicated an association with a reduced risk of IBD (Kostic et al., 2014). It’s important to note that BMI doesn’t accurately reflect fat mass and fat-free mass composition (Nevill et al., 2006). Several adipokines, secreted by adipocytes, significantly influence gut microbiota and modulate inflammation in the gastrointestinal milieu (Karrasch and Schaeffler, 2016). The distribution of fat accumulation in the body, combined with genetic factors, contributes to the balance of pro-inflammatory and anti-inflammatory adipokine cycles (Milajerdi et al., 2018).

The balance between these adipokines, particularly adiponectin and leptin, plays a crucial role in stimulating or inhibiting intestinal inflammation. Therefore, further research is needed to discover the exact association between body composition and fat accumulation and the risk of intestinal inflammation and IBD.

We found that several adverse lifestyles were associated with the development of IBD, among which smoking was confirmed as a risk factor for CD (class II evidence). Current studies have shown that smoking has become a recognized environmental risk factor for IBD (Soulakova et al., 2020). Studies have suggested that smoking doubles the risk of CD compared with non-smokers, but the risk is slightly reduced after quitting smoking (To et al., 2016). CD patients with smoking habits have a more complex disease course and are more likely to have invasive diseases (including the need for intestinal resection), increasing the risk of recurrence after surgery and resurgery (To et al., 2016; Lo Sasso et al., 2020; Soulakova et al., 2020). Passive smoking in childhood or prenatal maternal cigarette smoke inhalation increased the risk of CD, but no association with UC was found (Parkes et al., 2014). The mechanism of smoking and IBD is unclear, especially regarding the opposite effect of smoking in CD and UC. The effects of smoking on IBD are also thought to involve aspects such as loss of intestinal mucosal barrier integrity and potential epigenetic susceptibility. Finally, a large number of environmental factors are also risk or protective factors for IBD. Among them, this study found that people with personal toilets had a lower risk of CD (class III evidence), suggesting that environmental hygiene also plays a role in the occurrence and development of IBD. However, the exact mechanism is unclear.

### 4.2 Limitations and strengths

This study also has some limitations. First, we only searched databases in English and studies in other languages have been excluded, which may lead to potential bias. Second, this study only extracted published data, and those unpublished or forthcoming evidence-based evidence have been ignored. Third, this study directly extracted and analyzed existing data from systematic reviews and meta-analyses, and data from those original studies not included in systematic reviews and meta-analyses were not included. Another point to note is that in this umbrella review, no risk factors with Class I evidence were found. This may be related to the small sample size of most studies, unavoidable heterogeneity and publication bias. Therefore, the differences in the level of evidence need to be considered when interpreting the evidence in this article, which also needs to be further resolved by a large sample size and well-designed meta-analysis. Despite acknowledged limitations, this umbrella review provides the first comprehensive documentation of existing evidence from prior meta-analyses on risk factors for IBD. This umbrella review evaluated the advantages and disadvantages of existing evidence-based medical from systematic review and meta-analyses on the risk factors of IBD, helped to understand the potential risk factors for the occurrence and development of IBD in a more comprehensive way from multiple dimensions, provided a theoretical basis for the development of more clinical effective prevention and control measures for IBD, and provided directions for further clinical research. This study employed rigorous systematic methodologies. Two independent authors conducted literature searches, selected studies, and extracted data. When sufficient data were available, we reanalyzed RR, OR, WMD, or SMD using 95% CIs with random or fixed effects models. We thoroughly assessed heterogeneity and publication bias for each meta-analysis inclusion. Additionally, we utilized two established approaches, namely AMSTAR and evidence classification criteria, to appraise the methodological quality and evidence classification of each risk factor. This comprehensive evaluation enabled us to assess our confidence in the provided estimates.

## 5 Conclusion

This umbrella review extracted 92 risk factors that were significantly associated with IBD, including 62 risk factors and 30 protective factors, most of which were related to underlying diseases, personal lifestyle and environmental factors. After evaluating the quality of the evidence, only 10 risk factors were rated as class II and class III evidence in this study, including appendectomy, asthma (UC), asthma (CD), autism spectrum disorder, BMI, helicobacter pylori infection, hidradenitis suppurative (UC), hidradenitis suppurative (CD), personal toilets, and smoking. The findings in this paper help to develop better prevention and treatment measures to reduce the incidence of IBD, delay its progression, and reduce the burden of IBD-related disease worldwide.

## Data Availability

The original contributions presented in the study are included in the article/**Supplementary Material**. Further inquiries can be directed to the corresponding author.

## Supplementary material

Supplementary Table 1 Risk factors for IBD.

Supplementary Table 2 Assessments of AMSTAR scores.
