# Supplementary material for: Risk factors for inflammatory bowel disease: an umbrella review
Source: Front Cell Infect Microbiol. 2025 Jan 24;14:1410506. doi: 10.3389/fcimb.2024.1410506 (PMC11802543; doi:10.3389/fcimb.2024.1410506)
Supplement: Supplementary Table 1 — Risk factors for IBD. [file DataSheet1.docx]

**Supplementary materials**

**Supplementary Table S1**. Risk factors for IBD.

| **Risk factors** | **Exposure contrast** | **Diseases** | **Total eligible MA** | **Included MA** | **No. of cases/total** | **MA metric** | **Estimates, 95% CI** | **No. of studies**  **T/R/C/P** | **Effects model** | **I^2^; Q test P value** | **Egger test P value** | **AMSTAR** | **Evidence classification** |
| --- | --- | --- | --- | --- | --- | --- | --- | --- | --- | --- | --- | --- | --- |
| ***Significant associations*** | | | | | | | | | | | | | |
| Antibiotic use | Any vs. none | CDwest | 3 | Zhao2021 | NA/6,024^a,b^ | OR | 1.90, 1.60-2.27 | 18/0/5/13 | Random | 84.1;0 | 0.148 | 8 | IV |
| Antibiotic use | Any vs. none | UCwest | 3 | Zhao2021 | NA/4,996^a,b^ | OR | 1.40, 1.12-1.76 | 15/0/5/10 | Random | 86;0 | 0.884 | 8 | IV |
| Appendectomy | Yes vs. no | CD | 3 | Zhang2023 | 2,751/2,240,604^a^ | RR | 2.28, 1.66-3.14 | 6/0/6/0 | Random | 97.4;0.000 | 0.548 | 9 | II |
| Asthma | Yes vs. no | CD | 1 | Kuenzig2018 | 18,083/NA^a,b^ | RR | 1.31, 1.16-1.47 | 15/0/3/12 | Random | 88;<0.01 | NA | 9 | II |
| Asthma | Yes vs. no | UC | 1 | Kuenzig2018 | 18,329/NA^a,b^ | RR | 1.30, 1.21-1.40 | 16/0/3/13 | Random | 93;<0.01 | NA | 9 | II |
| Atopic dermatitis | Yes vs. no | UC | 2 | Lee 2020 | 266/74,380a | OR | 1.54, 1.07-2.18 | 3/0/3/0 | Random | 65; 0.06 | NA | 8 | IV |
| Autism spectrum disorder | Yes vs. no | IBD | 1 | Kim2022 | 23,506/115,297^a,b^ | OR | 1.57, 1.28-1.930 | 6/0/1/5 | Random | 87.1;<0.001 | NA | 9 | II |
| Bariatric Surgery | Before vs. after | IBD | 1 | Kermansaravi2022 | 708/149,385^a^ | OR | 1.17, 1.06-1.29 | 4/0/4/0 | Random | 90.2;0.000 | 0.677 | 9 | IV |
| BMI | Highest vs. lowest | IBD | 2 | Milajerdi2022 | NA/1,503,262^a^ | OR | 0.76, 0.66-0.88 | 5/0/5/0 | Random | 93.2;0.000 | NA | 8 | III |
| Breast feeding | Yes vs. no | CDwest | 4 | Zhao2021 | NA/7,011^a,b^ | OR | 0.87, 0.76-1.00 | 24/0/4/0 | Random | 74.9;0 | 0.041 | 8 | IV |
| Breast feeding | Yes vs. no | CDeast | 4 | Zhao2021 | NA/228^a,b^ | OR | 0.29, 0.11-0.81 | 2/0/0/2 | Random | 78.9;0.007 | 0.041 | 8 | IV |
| Campylobacter species | Yes vs. no | IBD | 2 | Castaño-Rodríguez2017 | 519/NA^b^ | OR | 2.969, 1.330-6.626 | 9/0/0/9 | Random | 82.03；0.008 | 0.104 | 7 | IV |
| Ceasarean | Yes vs. no | CDwest | 3 | Zhao2021 | NA/4892^a,b^ | OR | 1.16, 1.05-1.28 | 9/0/1/8 | Random | 18.8;0.270 | NA | 8 | IV |
| Ceasarean | Yes vs. no | UCwest | 3 | Zhao2021 | NA/2131^a,b^ | OR | 1.17, 1.08-1.27 | 7/0/1/6 | Random | 0; 0.493 | NA | 8 | IV |
| Cholesterol | Highest vs. lowest | UCeast | 1 | Zhao2021 | NA/307^b^ | OR | 1.66, 1.26-2.20 | 4/0/0/4 | Random | 0；0.767 | NA | 8 | IV |
| Chronic obstructive pulmonary diseases | Yes vs. no | CD | 1 | Labarca2019 | NA/675^a^ | RR | 2.29, 1.51-3.48 | 4/0/4/0 | Random | 62;0.05 | NA | 10 | IV |
| Chronic obstructive pulmonary diseases | Yes vs. no | UC | 1 | Labarca2019 | NA/680^a^ | RR | 1.79, 1.39-2.29 | 4/0/4/0 | Random | 19;0.30 | NA | 10 | IV |
| Chronic obstructive pulmonary diseases | Yes vs. no | CD/UC | 1 | Labarca2019 | NA/1355^a^ | RR | 2.02, 1.56-2.63 | 4/0/4/0 | Random | 72;0.0008 | NA | 10 | IV |
| Coffee | Highest vs. lowest | UCwest | 3 | Zhao2021 | NA/1,072^b^ | OR | 0.58, 0.40-0.83 | 6/0/0/6 | Random | 75.9;0 | NA | 8 | IV |
| Coffee | Highest vs. lowest | UCeast | 3 | Zhao2021 | NA/306^b^ | OR | 0.53, 0.39-0.73 | 3/0/0/3 | Random | 0;0.501 | NA | 8 | IV |
| Contact with farm animals | Yes vs. no | CDwest | 2 | Zhao2021 | NA/1137^b^ | OR | 0.70, 0.54-0.91 | 4/0/0/4 | Random | 68.2；0.001 | NA | 8 | IV |
| Dietary fat | High vs. low | IBD,UC,CD | 2 | Wu2016 | 1,084/398,081^a,b^ | ES | 1.52, 1.16-1.99 | 8/0/3/5 | Random | 0；0.527 | 0.728 | 8 | IV |
| Dietary fiber intake | Highest vs. lowest | IBD | 4 | Milajerdi2021 | NA/2,758^a,b^ | RR | 0.83, 0.70-0.97 | 6/0/3/3 | fixed | 45.6;0.037 | NA | 8 | IV |
| Dietary fiber intake | Highest vs. lowest | CD | 4 | Milaherdi2021 | NA/2,518^a,b^ | ES | 0.59, 0.46-0.74 | 5/0/3/2 | fixed | 0;0.869 | NA | 8 | IV |
| Dietary fiber intake | Highest vs. lowest | CD | 4 | Milaherdi2021 | NA/2,518^a,b^ | ES | 0.59, 0.46-0.74 | 5/0/3/2 | fixed | 0;0.869 | NA | 8 | IV |
| Egg | Highest vs. lowest | UCeast | 1 | Zhao2021 | NA/613^b^ | OR | 1.37, 1.12-1.68 | 6/0/0/6 | Random | 0.8;0.436 | 0.189 | 8 | IV |
| Exposure to pets | Yes vs. no | CDeast | 2 | Zhao2021 | NA/460^a^ | OR | 0.54, 0.38-0.77 | 3/0/0/3 | Random | 68.0;0.002 | 0.42 | 8 | IV |
| Exposure to pets | Yes vs. no | UCwest | 2 | Zhao2021 | NA/1966^b^ | OR | 0.73, 0.60-0.88 | 11/0/0/11 | Random | 62.8；0 | 0.277 | 8 | IV |
| Exposure to pets | Yes vs. no | UCeast | 2 | Zhao2021 | NA/848^b^ | OR | 0.66, 0.47-0.93 | 3/0/0/3 | Random | 72.9；0 | 0.277 | 8 | IV |
| Exposure to pets | Yes vs. no | CDwest | 2 | Zhao2021 | NA/529^b^ | OR | 0.54, 0.38-0.77 | 3/0/0/3 | Random | 22.7;0.248 | NA | 8 | IV |
| Fatty acids | Highest vs. lowest | UCeast | 1 | Zhao2021 | NA/253^b^ | OR | 1.43,1.19-1.72 | 3/0/0/3 | Random | 16.3;0.226 | 0.02 | 8 | IV |
| Fiber intake | Per 10 g increment/day | CD | 3 | Zeng2017 | NA/580^a,b^ | RR | 0.853, 0.762-0.955 | 4/0/1/3 | Random | 0;0.730 | 0.708 | 8 | IV |
| Fruit | Highest vs. lowest | UC | 5 | Milajerdi2021 | NA/2,154^a^ | RR | 0.69, 0.55-0.86 | 4/0/4/0 | fixed | 87.0;0.000 | NA | 8 | IV |
| Fruit | Highest vs. lowest | CD | 5 | Milajerdi2021 | NA/2154^a^ | RR | 0.47,0.38-0.58 | 4/0/4/0 | fixed | 32.1；0.220 | NA | 8 | IV |
| Fruit | Highest vs. lowest | IBD | 5 | Milajerdi2021 | NA/2154^a^ | RR | 0.56, 0.48-0.65 | 4/0/4/0 | fixed | 79.0;0.000 | NA | 8 | IV |
| HCMV infection | Yes vs. no | I BD | 1 | Lv2017 | 135/529b | OR | 4.99, 2.40-10.40 | 12/0/0/12 | fixed | 66;<0.0001 | NA | 8 | IV |
| Healthy/prudent dietary patterns | Yes vs. no | CD | 1 | Khorshidi2020 | NA/NA^a,b^ | OR | 0.39, 0.16-0.62 | 5/0/2/3 | Random | 67.9;0.014 | 0.142 | 8 | IV |
| Helicobacter pylori | Yes vs. no | IBD | 8 | Bouriat2022 | 2,545/NA^b^ | OR | 0.42, 0.32-0.56 | 19/0/0/19 | Random | NA;<0.0001 | NA | 8 | II |
| Hidradenitis suppurativa | Yes vs. no | CD | 2 | Phan2020 | NA/101,940b | OR | 2.25, 1.52-3.32 | 5/0/0/5 | Random | 92;<0.00001 | NA | 10 | III |
| Hidradenitis suppurativa | Yes vs. no | UC | 2 | Phan2020 | NA/39,455b | OR | 1.56, 1.26-1.94 | 3/0/0/3 | Random | 36;0.21 | NA | 10 | II |
| Hidradenitis suppurativa | Yes vs. no | Unspecified IBD | 2 | Phan2020 | NA/9,221b | OR | 2.80, 1.81-4.35 | 2/0/0/2 | Random | 67;0.08 | NA | 10 | IV |
| IBD family history | Yes vs. no | UCwest | 1 | Zhao2021 | NA/3,880^b^ | OR | 3.64, 2.85-4.63 | 18/0/1/17 | Random | 52.5;0.003 | 0 | 8 | IV |
| IBD family history | Yes vs. no | UCeast | 1 | Zhao2021 | NA/1,281^b^ | OR | 3.88, 2.54-5.94 | 6/0/0/6 | Random | 0;0425 | 0 | 8 | IV |
| IBD family history | Yes vs. no | CDwest | 1 | Zhao2021 | NA/6,369^b^ | OR | 4.09, 3.49-4.81 | 25/0/1/24 | Random | 36.1;0.029 | 0 | 8 | IV |
| IBD family history | Yes vs. no | CDeast | 1 | Zhao2021 | NA/523^b^ | OR | 4.39, 2.55-7.59 | 4/0/0/4 | Random | 0;0.726 | 0 | 8 | IV |
| Isotretinoin | Yes vs. no | UCwest | 2 | Zhao2021 | NA/14,214^a,b^ | OR | 1.48, 1.06-2.07 | 5/0/1/4 | Random | 41.8;0.127 | NA | 8 | IV |
| Meat and meat product | Highest vs. lowest | CDwest | 3 | Zhao2021 | NA/1,419^a,b^ | OR | 1.58, 1.20-2.08 | 2007/1/6 | Random | 83.5;0 | NA | 8 | IV |
| Milk and dairy products | Highest vs. lowest | CDwest | 1 | Zhao2021 | NA/1077^a,b^ | OR | 0.79, 0.65-0.97 | 8/0/1/7 | Random | 64.6；0 | 0.281 | 8 | IV |
| More physical activity | Yes vs. no | CDwest | 1 | Zhao2021 | NA/1,055^a,b^ | OR | 0.67, 0.55-0.83 | 8/0/2/6 | Random | 54.5;0.004 | 0.028 | 8 | IV |
| More physical activity | Yes vs. no | CDeast | 1 | Zhao2021 | NA/1,625^a,b^ | OR | 0.73, 0.58-0.93 | 3/0/1/2 | Random | 62.9;0.019 | 0.028 | 8 | IV |
| More physical activity | Yes vs. no | UCeast | 1 | Zhao2021 | NA/934^b^ | OR | 0.63, 0.51-0.78 | 2/0/0/2 | Random | 21.5;0.281 | 0.289 | 8 | IV |
| MUFA | Highest vs. lowest | UCeast | 3 | Zhao2021 | NA/253^b^ | OR | 1.66, 1.19-2.32 | 3/0/1/2 | Random | 22.6;0.242 | NA | 8 | IV |
| Multiple birth | Yes vs. no | CDwest | 2 | Zhao2021 | NA/14,758^a,b^ | OR | 0.76, 0.60-0.97 | 10/0/1/9 | Random | 68.6;0 | 0.619 | 8 | IV |
| Multiple birth | Yes vs. no | UCeast | 2 | Zhao2021 | NA/1,273^b^ | OR | 0.79, 0.66-0.95 | 2/0/0/2 | Random | 0;0.393 | 0.625 | 8 | IV |
| Multiple sclerosis | Yes vs. no | UC | 1 | Wang2022 | 127/1,280b | RR | 1.42, 1.17-1.71 | NA | Fixed | 38;0.0003 | 0.196 | 8 | IV |
| Multiple sclerosis | Yes vs. no | CD | 1 | Wang2022 | 107/977b | RR | 1.41, 1.14-1.74 | NA | Fixed | 0;0.001 | 0.196 | 8 | IV |
| Multiple sclerosis | Yes vs. no | UC and CD | 1 | Wang2022 | 234/2,267b | RR | 1.41, 1.23-1.63 | NA | Fixed | 0;<0.00001 | 0.196 | 8 | IV |
| n-6PUFA | Highest vs. lowest | UCwest | 1 | Zhao2021 | NA/533^a,b^ | OR | 1.25, 1.01-1.54 | 4/0/0/1 | Random | 32.5;0.139 | NA | 8 | IV |
| Number of siblings | High vs. low | CD | 1 | Cholapranee2016 | NA/13,185^b^ | OR | 0.93, 0.88-0.98 | 5/0/0/5 | Random | NR | NR | 8 | IV |
| Oral contraceptive | Yes vs. no | CDwest | 4 | Zhao2021 | NA/4,852^a,b^ | OR | 1.31, 1.12-1.53 | 24/0/2/22 | Random | 54.2;0 | 0.034 | 8 | IV |
| Oral contraceptive | Yes vs. no | UCweat | 4 | Zhao2021 | NA/4,045^a,b^ | OR | 1.18, 1.03-1.35 | 19/0/1/18 | Random | 0;0.877 | 0.97 | 8 | IV |
| Otitis media infection | Yes vs. no | IBD | 1 | Agrawal2021 | NA/17967^a^ | OR | 2.11, 1.22-3.62 | 2/0/2/0 | Random | 36.9;0.21 | NA | 9 | IV |
| Otitis media infection | Yes vs. no | CD | 1 | Agrawal2021 | NA/10,318^a^ | OR | 1.95, 1.20-3.17 | 2/0/2/0 | Random | 4.41;0.31 | NA | 9 | IV |
| Periodontal | Yes vs. no | IBD | 2 | Lorenzo-Pouso2021 | 657/NA^a,b^ | RR | 2.78, 1.36-5.69 | 8/0/1/7 | Random | 87.89;0.00001 | 0.008 | 9 | IV |
| Periodontal | Yes vs. no | CD | 2 | Lorenzo-Pouso2021 | 619/NA^a,b^ | RR | 3.41, 1.36-8.56 | 7/0/1/6 | Random | 946;0.0001 | 0.008 | 9 | IV |
| Periodontal | Yes vs. no | UC | 2 | Lorenzo-Pouso2021 | 409/NA^a,b^ | RR | 3.98, 2.02-7.87 | 7/0/1/6 | Random | 72.88;000001 | 0.008 | 9 | IV |
| Personal toilet | Yes vs. no | UC | 1 | Cholapranee2016 | NA/20,365^a^ | OR | 0.73, 0.59-0.88 | 9/0/1/8 | Random | NA | <0.05 | 8 | III |
| Poliomyelitis | Yes vs. no | CD | 1 | Pineton2015 | NA/345^b^ | RR | 2.28, 1.12-4.68 | 2/0/0/2 | Random | 0;0.606 | NA | 8 | IV |
| Poliomyelitis | Yes vs. no | UC | 1 | Pineton2015 | NA/174^b^ | RR | 3.48, 1.24-9.71 | 2/0/0/2 | Random | 0;0.5397 | NA | 8 | IV |
| Proton pump inhibitors, PPIs | Any vs. none | IBD | 1 | Shastri2022 | NA/12,714^a,b^ | OR | 2.43, 1.18-5.02 | 6/0/1/5 | Random | 86;0.00001 | NA | 9 | IV |
| Psoriasis | Yes vs. no | CD | 1 | Fu2018 | NA/85,761^a^ | RR | 2.53, 1.65-3.89 | 4/0/4/0 | Random | 55;0.08 | NA | 8 | IV |
| Psoriasis | Yes vs. no | UC | 1 | Fu2018 | NA/85,761^a^ | RR | 1.71, 1.55-1.89 | 4/0/4/0 | Random | 0;0.55 | NA | 8 | IV |
| Rosacea | Yes vs. no | IBD | 2 | Han2019 | NAc | ES | 1.32, 1.18-1.49 | NA | Random | 31.6;0.187 | >0.05 | 8 | IV |
| Smoking | Any vs. no | CDwest | 4 | Zhao2021 | NA/10021^a,b^ | OR | 1.61, 1.47-1.77 | 53/0/4/49 | Random | 64.4;0 | 0.017 | 8 | II |
| Smoking | Any vs. no | CDeast | 4 | Zhao2021 | NA/1550^a,b^ | OR | 1.29, 1.09-1.52 | 11/0/1/10 | Random | 49.2;0.008 | 0.017 | 8 | IV |
| Soft drink | Highest vs. lowest | CDwest | 3 | Zhao2021 | NA/1,735^a,b^ | OR | 1.56, 1.25-1.95 | 8/0/1/7 | Random | 56.7;0.002 | 0.965 | 8 | IV |
| Suger | Highest vs. lowest | UC | 2 | Khademi2021 | NA/1,213^a,b^ | RR | 1.59, 1.15-2.20 | 4/0/2/2 | Random | 0;0.58 | NA | 8 | IV |
| Suger | Highest vs. lowest | CD | 2 | Khademi2021 | NA/1,213^a,b^ | RR | 1.90, 1.06-3.14 | 4/0/2/2 | Random | 57.5;0.051 | NA | 8 | IV |
| Suger | Highest vs. lowest | IBD | 2 | Khademi2021 | NA/1,213^a,b^ | RR | 1.71, 1.24-2.38 | 4/0/2/2 | Random | 41.5;0.14 | NA | 8 | IV |
| Tea consumption | Highest vs. lowest | CD | 3 | Yang2019 | NA/288^b^ | RR | 0.70, 0.53-0.93 | 2/0/0/2 | Random | 0；0.636 | NA | 8 | IV |
| Tea consumption | Highest vs. lowest | UC | 3 | Nie2017 | NA/1,741^b^ | RR | 0.69, 0.58-0.83 | 3/0/0/3 | Random | 0；0.697 | 0.623 | 8 | IV |
| Tonsillectomy | Yes vs. no | CDwest | 2 | Zhao2021 | NA/28,971^a,b^ | OR | 1.27, 1.09-1.46 | 14/0/1/13 | Random | 66.8;0 | 0.39 | 8 | IV |
| Total energy | Highest vs. lowest | CDwest | 1 | Zhao2021 | NA/227b | OR | 1.44, 1.10-1.90 | 2/0/0/2 | Random | 0;0.703 | NA | 8 | IV |
| Urban living | Yes vs. no | CDwest | 4 | Zhao2021 | NA/3,360^b^ | OR | 1.42, 1.28-1.56 | 27/0/0/27 | Random | 86.2;0 | 0.071 | 8 | IV |
| Urban living | Yes vs. no | UCwest | 4 | Zhao2021 | NA/2,436^b^ | OR | 1.22,1.11-1.34 | 4/0/0/4 | Random | 0;0.773 | 0.26 | 8 | IV |
| Vegetables | Highest vs. lowest | CDwest | 5 | Zhao2021 | NA/1,033^a,b^ | OR | 0.67, 0.57-0.79 | 10/0/3/7 | Random | 43.2;0019 | 0.041 | 8 | IV |
| Vitamin D | Highest vs. lowest | CDwest | 5 | Zhao2021 | NA/684^b^ | OR | 1.58, 1.17-2.13 | 13/0/0/13 | Random | 27.3;0.169 | 0.029 | 8 | IV |
| Vitamin D | Highest vs. lowest | UCwest | 5 | Zhao2021 | NA/380^b^ | OR | 1.98, 1.17-3.37 | 8/0/0/8 | Random | 59.8;0.015 | 0.252 | 8 | IV |
| Vitamin D | Highest vs. lowest | UCeast | 5 | Zhao2021 | NA/121^b^ | OR | 2.20, 1.30-3.72 | 3/0/0/3 | Random | 0;0.414 | 0.252 | 8 | IV |
| Western dietary pattern | Yes vs. no | CD | 1 | Li2020 | NA/707^a,b^ | RR | 1.72, 1.01-2.93 | 7/0/3/4 | Random | 74.8;0.000 | NA | 8 | IV |
| Western dietary pattern | Yes vs. no | UC | 1 | Li2020 | NA/683^a,b^ | RR | 2.15, 1.38-3.34 | 8/0/3/5 | Random | 65.9;0.005 | NA | 8 | IV |
| Western dietary pattern | Yes vs. no | IBDCD and UC | 1 | Li2020 | NA/1390^a,b^ | RR | 1.92, 1.37-2.68 | 8/0/3/5 | Random | 70.4;0.000 | NA | 8 | IV |
| ***Non-significant associations*** | | | | | | | | | | | | | |
| 35older maternal age ≥35 years | Yes vs. no | IBD | 1 | Agrawal2021 | NA/NA^a,b^ | OR | 0.86, 0.45-1.65 | 4/0/2/2 | Random | 87.05;<0.001 | NA | 9 | NS |
| 35older maternal age ≥35 years | Yes vs. no | CD | 1 | Agrawal2021 | NA/NA^a^ | OR | 077, 0.22-2.73 | 2/0/2/0 | Random | 95.27;<0.001 | NA | 9 | NS |
| Alcohol | Highest vs. lowest | UC | 2 | Nie2017 | NA/2386^a,b^ | RR | 0.95, 0.65-1.39 | 9/0/3/6 | Random | 66.9；0.002 | 0.03 | 8 | NS |
| Alcohol | Highest vs. lowest | CD | 2 | Yang2019 | NA/732^a,b^ | RR | 0.85, 0.68-1.08 | 6/0/2/4 | Random | 0;0.453 | 0.992 | 8 | NS |
| Antibiotic use | Highest vs. lowest | CDeast | 3 | Zhao2021 | NA/356^b^ | OR | 0.55, 0.29-1.01 | 3/0/0/3 | Random | 78.1;0.001 | 0.148 | 8 | NS |
| antibiotic use | Highest vs. lowest | UCeast | 3 | Zhao2021 | NA/1,176^b^ | OR | 0.85, 0.56-1.27 | 4/0/0/4 | Random | 79;0 | 0.884 | 8 | NS |
| Atopic dermatitis | Yes vs. no | CD | 2 | Lee 2020 | 118/74,380a | OR | 1.14, 0.60-2.15 | 3/0/3/0 | Random | 83; <0.01 | NA | 8 | NS |
| Bed or bedroom sharing | Yes vs. no | UCwest | 2 | Zhao2021 | NA/496^b^ | OR | 0.68, 0.44-1.03 | 4/0/0/4 | Random | 82.2;0 | NA | 8 | NS |
| Bed or bedroom sharing | Yes vs. no | UCeast | 2 | Zhao2021 | NA/592^b^ | OR | 0.88, 0.33-2.33 | 2/0/0/2 | Random | 89.7;0 | NA | 8 | NS |
| Bed or bedroom sharing | Yes vs. no | CDwest | 2 | Zhao2021 | NA/896^b^ | OR | 0.73, 0.49-1.09 | 7/0/0/7 | Random | 80.6;0 | NA | 8 | NS |
| BGG vaccine | Yes vs. no | IBD | 2 | Agrawal2021 | NA/NA^a^ | OR | 1.549, 0.969-2.477 | 4/0/4/0 | Random | 37.58;0.19 | NA | 9 | NS |
| BGG vaccine | Yes vs. no | CD | 2 | Agrawal2021 | NA/NA^a^ | OR | 1.773, 0.846-3.714 | 3/0/3/0 | Random | 53.17;0.12 | NA | 9 | NS |
| BMI | Highest vs. lowest | CD | 2 | Milajerdi2022 | NA/503,262^a^ | RR | 0.87, 0.73-1.05 | 5/0/5/0 | Random | 88.9;0.000 | NA | 8 | NS |
| Coffee | Highest vs. lowest | CDwest | 3 | Zhao2021 | NA/748^b^ | OR | 0.72, 0.47-1.11 | 5/0/0/5 | Random | 73.9;0 | NA | 8 | NS |
| Dietary fiber intake | Highest vs. lowest | UC | 4 | Milaherdi2021 | NA/2,779^a,b^ | ES | 1.09, 0.88-1.34 | 6/0/4/2 | fixed | 0;0.448 | NA | 8 | NS |
| Diphteria and tetanus containing vaccines | Yes vs. no | IBD | 1 | Pineton2015 | NA/524^b^ | RR | 1.24, 0.80-1.94 | 3/0/0/3 | Random | 0;0.97682 | NA | 8 | NS |
| Egg | Highest vs. lowest | CDwest | 1 | Zhao2021 | NA/459^a,b^ | OR | 1.02, 0.82-1.27 | 5/0/1/4 | Random | 6.4;0.379 | NA | 8 | NS |
| Egg | Highest vs. lowest | CDeast | 1 | Zhao2021 | NA/396^b^ | OR | 1.38, 0.97-1.99 | 4/0/0/4 | Random | 44.2;0.111 | NA | 8 | NS |
| EHS | Yes vs. no | IBD | 3 | Castaño-Rodríguez2017 | 1,812/NA^b^ | OR | 1.511, 0.948-2.406 | 14/0/0/14 | Random | 57.37;0.083 | 0.584 | 7 | NS |
| Exposure to pets | Yes vs. no | CDWest | 2 | Zhao2021 | NA/3,003^a^ | OR | 0.98, 0.81-1.19 | 14/0/0/14 | Random | 73.5;0 | 0.42 | 8 | NS |
| Fat | Highest vs. lowest | CDwest | 4 | Zhao2021 | NA/724^a,b^ | OR | 1.09, 0.94-1.27 | 6/0/3/3 | Random | 0;0955 | NA | 8 | NS |
| Fat | Highest vs. lowest | UCwest | 4 | Zhao2021 | NA/778^a,b^ | OR | 1.03, 0.91-1.17 | 6/0/3/3 | Random | 0;0.833 | 0.01 | 8 | NS |
| Fat | Highest vs. lowest | UCeast | 4 | Zhao2021 | NA/307^b^ | OR | 1.41, 0.98-2.02 | 4/0/0/4 | Random | 34.2;0.134 | 0.01 | 8 | NS |
| Fatty acids | Highest vs. lowest | CDwest | 1 | Zhao2021 | NA/542^a,b^ | OR | 1.06, 0.95-1.20 | 4/0/2/2 | Random | 22.1;0.101 | NA | 8 | NS |
| Fatty acids | Highest vs. lowest | UCwest | 1 | Zhao2021 | NA/797^a,b^ | OR | 0.99, 0.91-1.08 | 7/0/2/5 | Random | 8.8；0.304 | 0.02 | 8 | NS |
| Fiber intake | Highest vs. lowest | UC | 3 | Wang2017 | NA/827^a,b^ | RR | 1.001, 0.971，1.032 | 5/0/2/3 | Random | 0;0.634 | 0.552 | 8 | NS |
| Fish | Highest vs. lowest | IBD | 2 | Mozaffari2020 | NA/823^b^ | ES | 0.68, 0.46-1 | 5/0/0/5 | Random | 76.4;0.001 | 0.08 | 8 | NS |
| Fruits | Highest vs. lowest | UCwest | 5 | Zhao2021 | NA/1,316^a,b^ | OR | 0.82, 0.65-1.02 | 8/0/3/5 | Random | 76.5;0 | 0.981 | 8 | NS |
| Hot water | Highest vs. lowest | CD | 1 | Cholapranee2016 | NA/1,444^a,b^ | OR | 1.0, 0.73-1.27 | 10/0/1/9 | Random | NA | NA | 8 | NS |
| Hot water | Highest vs. lowest | UC | 1 | Cholapranee2016 | NA/3,081^a,b^ | OR | 0.98, 0.63-1.34 | 9/0/1/8 | Random | NA | NA | 8 | NS |
| Isotretinoin | Yes vs. no | CDwest | 2 | Zhao2021 | NA/17,605^a,b^ | OR | 0.93, 0.68-1.28 | 5/0/1/4 | Random | 44.8;0.107 | NA | 8 | NS |
| Job strain | Yes vs. no | CD | 1 | Heikkilä2014 | NA/126^a^ | HR | 0.89, 0.52-1.52 | 11/0/11/0 | Random | 0; 0.9 | NA | 8 | NS |
| Job strain | Yes vs. no | UC | 1 | Heikkilä2014 | NA/414^a^ | HR | 1.14, 0.80-1.61 | 11/0/11/0 | Random | 27.1; 0.2 | NA | 8 | NS |
| Low birth weight | Yes vs. no | IBD | 4 | Agrawal2021 | NA/4,022,451^a,b^ | pOR | 0.92, 0.72-1.16 | 10/0/4/6 | Random | 76.75;<0.001 | 0.353 | 9 | NS |
| Low birth weight | Yes vs. no | CD | 4 | Agrawal2021 | NA/1,901,512^a,b^ | pOR | 0.86, 0.62-1.23 | 6/0/2/4 | Random | 70.72;0.004 | 0.353 | 9 | NS |
| Low birth weight | Yes vs. no | UC | 4 | Agrawal2021 | NA/438,714^a,b^ | pOR | 083, 0.41-1.68 | 3/0/0/3 | Random | 81.66;0.004 | 0.353 | 9 | NS |
| Measles vaccine | Yes vs. no | IBD | 2 | Agrawal2021 | NA^a^ | OR | 1.078, 0.911-1.275 | 12/0/12/0 | Random | 55.52；0.004 | 0.206 | 9 | NS |
| Measles vaccine | Yes vs. no | CD | 2 | Agrawal2021 | NA^a^ | OR | 1.06, 0.822-1.366 | 9/0/9/0 | Random | 64.69；0.004 | NA | 9 | NS |
| Measles vaccine | Yes vs. no | UC | 2 | Agrawal2021 | NA^a^ | OR | 1.046, 0.844-1.296 | 6/0/6/0 | Random | 28.20；0.22 | NA | 9 | NS |
| Meat and meat product | Highest vs. lowest | UCwest | 3 | Zhao2021 | NA/820^a,b^ | OR | 1.46, 0.88-2.44 | 4/0/1/3 | Random | 80.3;0 | 0.037 | 8 | NS |
| Meat and meat product | Highest vs. lowest | UCeast | 3 | Zhao2021 | NA/531^b^ | OR | 1.10, 0.98-1.24 | 6/0/0/6 | Random | 0;0.506 | 0.037 | 8 | NS |
| Milk and dairy products | Highest vs. lowest | CDeast | 1 | Zhao2021 | NA/177^b^ | OR | 0.84, 0.45-1.57 | 2/0/0/2 | Random | 68.4;0.023 | 0.281 | 8 | NS |
| Milk and dairy products | Highest vs. lowest | UCeast | 1 | Zhao2021 | NA/1,611^b^ | OR | 1.02, 0.83-1.24 | 5/0/0/5 | Random | 22.2;0.219 | 0.777 | 8 | NS |
| Milk and dairy products | Highest vs. lowest | UCwest | 1 | Zhao2021 | NA/681^a,b^ | OR | 0.81, 0.65-1.03 | 5/0/1/4 | Random | 50.5;0.04 | 0.777 | 8 | NS |
| More physical activity | Yes vs. no | UCwest | 1 | Zhao2021 | NA/1,008^a,b^ | OR | 0.84, 0.70-1.01 | 6/0/2/4 | Random | 49.6;0.018 | 0.289 | 8 | NS |
| MUFA | Highest vs. lowest | CDwest | 3 | Zhao2021 | NA/472^a.b^ | OR | 1.09, 0.91-1.29 | 3/0/1/2 | Random | 0；0.685 | NA | 8 | NS |
| MUFA | Highest vs. lowest | UCwest | 3 | Zhao2021 | NA/629^a,b^ | OR | 1.00, 0.87-1.16 | 4/0/1/3 | Random | 0;0.95 | NA | 8 | NS |
| Multiple birth | Yes vs. no | UCwest | 2 | Zhao2021 | NA/17,335^b^ | OR | 0.90, 0.72-1.12 | 9/0/0/9 | Random | 56.9;0.013 | 0.625 | 8 | NS |
| n-3PUFAs | Highest vs. lowest | IBD | 2 | Mozaffari2020 | NA/1102^a,b^ | ES | 1.17, 0.80-1.72 | 5/0/3/2 | Random | 57.3；0.03 | 0.3 | 8 | NS |
| n-3PUFAs | Highest vs. lowest | UC | 2 | Mozaffari2020 | NA/634^a,b^ | ES | 0.96, 0.75-1.22 | 2/0/1/1 | fixed | 0;0.53 | 0.3 | 8 | NS |
| n-3PUFAs | Highest vs. lowest | CD | 2 | Mozaffari2020 | NA/468^a,b^ | ES | 1.62, 0.38-6.78 | 4/0/2/2 | Random | 80.1；0.02 | 0.3 | 8 | NS |
| n-6PUFA | Highest vs. lowest | CDwest | 1 | Zhao2021 | NA/472^a.b^ | OR | 1.02, 0.90-1.16 | 3/0/1/2 | Random | 0;0.527 | NA | 8 | NS |
| n-6PUFA | Highest vs. lowest | UCeast | 1 | Zhao2021 | NA/253^b^ | OR | 1.11, 0.75-1.63 | 3/0/0/3 | Random | 43.2；0.08 | NA | 8 | NS |
| Number of siblings | Highest vs. lowest | UC | 1 | Cholapranee2016 | NA/16,729^b^ | OR | 0.98, 0.94-1.03 | 6/0/0/6 | Random | NR | NR | 8 | NS |
| Number of siblings | Highest vs. lowest | IBD | 1 | Cholapranee2016 | NA/33,516^b^ | OR | 0.95, 0.84-1.06 | 2/0/0/2 | Random | NR | NR | 8 | NS |
| Older Maternal age≥35 years | Yes vs. no | IBD | 4 | Agrawal2021 | NA^a,b^ | OR | 0.863, 0.452-1.648 | 4/0/2/2 | Random | 87.05; <0.001 | NA | 9 | NS |
| Older Maternal age≥35 years | Yes vs. no | CD | 2 | Agrawal2021 | NA^a^ | OR | 0.773, 0.219-2.726 | 2/0/2/0 | Random | 95.27;<0.001 | NA | 9 | NS |
| Oral contraceptive | Yes vs. no | CDeast | 4 | Zhao2021 | NA/216^b^ | OR | 0.68, 0.42-1.11 | 3/0/0/3 | Random | 0;0.772 | 0.034 | 8 | NS |
| Oral contraceptive | Yes vs. no | UCeast | 4 | Zhao2021 | NA/2,109^b^ | OR | 0.90, 0.41-1.98 | 5/0/0/5 | Random | 84.2;0 | 0.97 | 8 | NS |
| Passive exposure to tobacco smoke | Yes vs. no | CD | 7 | Agrawal2021 | NA/5,985^b^ | OR | 1.084, 0.951-1.237 | 3/0/0/3 | Random | 64.16;0 | NA | 9 | NS |
| Personal toilet | Yes vs. no | CD | 1 | Cholapranee2016 | NA/16,488^a,b^ | OR | 1.11, 0.81-1.40 | 10/0/1/9 | Random | NA，， | <0.05 | 8 | NS |
| Poliomyelitis | Yes vs. no | IBD | 1 | Pineton2015 | NA/666^b^ | RR | 1.79, 0.88-3.66 | 3/0/0/3 | Random | 67;0.0494 | NA | 8 | NS |
| Premature birth | Yes vs. no | IBD | 8 | Agrawal2021 | NA^a,b^ | OR | 1.055, 0.933-1.194 | 8/0/2/6 | Random | 0;049 | 0.41 | 9 | NS |
| Premature birth | Yes vs. no | CD | 5 | Agrawal2021 | NA^a,b^ | OR | 1.073, 0.890-1.293 | 5/0/1/4 | Random | 5.33;0.38 | 0.41 | 9 | NS |
| Premature birth | Yes vs. no | UC | 5 | Agrawal2021 | NA^a,b^ | OR | 1.024, 0.669-1.570 | 5/0/1/4 | Random | 53.57;007 | 0.41 | 9 | NS |
| Premature birth | Yes vs. no | IBD | 1 | Agrawal2021 | NA/1,635,820^a,b^ | OR | 1.06, 0.93-1.19 | 9/0/2/7 | Random | 0;<0.001 | 0.41 | 9 | NS |
| Premature birth | Yes vs. no | CD | 1 | Agrawal2021 | NA/151,217^a,b^ | OR | 1.07, 0.89-1.29 | 5/0/1/4 | Random | 5.33;<0.001 | NA | 9 | NS |
| Premature birth | Yes vs. no | UC | 1 | Agrawal2021 | NA/153,669^a,b^ | OR | 1.02, 0.67-1.57 | 6/0/1/5 | Random | 53.57;<0.001 | NA | 9 | NS |
| Primary sclerosing cholangitis | Yes vs. no | IBD | 1 | Zhang2022 | 5,636/NA^c^ | OR | 1.12, 0.85-1.47 | 4 | Fixed | 34;0.21 | NA | 8 | NS |
| Protein intake | Highest vs. lowest | UC/IBD | 5 | Zhou2022 | NA^a^ | RR | 1.56, 0.38-6.35 | 2/0/2/0 | Random | 86.4；0.007 | 0.327 | 8 | NS |
| PUFAs Polyunsaturated fat intake | Per 30g increment/day in total fat intake | UC | 2 | Wang2016 | NA^d^ | RR | 1.247, 0.948-1.640 | 4/NA | random | 25.4；0.259 | NA | 8 | NS |
| Smoking | Any vs. none | UCwest | 4 | Zhao2021 | NA/10,491^a,b^ | OR | 0.92, 0.79-1.08 | 52/0/6/46 | Random | 89.4;0 | 0.96 | 8 | NS |
| Smoking | Any vs. none | UCeast | 4 | Zhao2021 | NA/5,397^a,b^ | OR | 0.92, 0.71-1.20 | 17/0/0/0 | Random | 82.8;0.008 | 0.96 | 8 | NS |
| Soft drink | Highest vs. lowest | CDeast | 3 | Zhao2021 | NA/450^a,b^ | OR | 0.86, 0.46-1.59 | 2/0/0/2 | Random | 0;0.381 | 0.965 | 8 | NS |
| Soft drink | Highest vs. lowest | UCwest | 3 | Zhao2021 | NA/1,276^a.b^ | OR | 1.13, 0.98-1.32 | 5/0/1/4 | Random | 32.9;0.127 | NA | 8 | NS |
| Soft drink | Highest vs. lowest | UCeast | 3 | Zhao2021 | NA/411^b^ | OR | 1.34, 0.83-2.17 | 3/0/0/3 | Random | 43.8;0.148 | NA | 8 | NS |
| Statin use | Highest vs. lowest | IBD | 1 | Bhagavathula2021 | NA^a,b^ | HR | 0.81, 0.63-1.06 | 5/0/2/3 | Random | 81.3；<0.001 | 0.01 | 8 | NS |
| Statin use | Highest vs. lowest | CD | 1 | Bhagavathula2021 | NA^a,b^ | HR | 0.94, 0.72-1.25 | 3/0/2/1 | Random | 85.9;<0.001 | 0.01 | 8 | NS |
| Statin use | Highest vs. lowest | UC | 1 | Bhagavathula2021 | NA^a,b^ | HR | 0.89, 0.70-1.12 | 3/0/2/1 | Random | 92.5;<0.001 | 0.01 | 8 | NS |
| Sugar-Sweetened Beverages Consumption | Highest vs. lowest | UC | 1 | Khademi2021 | NA/1,622^a,b^ | RR | 1.02, 0.92-1.12 | 3/0/1/2 | Random | 14.4;032 | NA | 8 | NS |
| Sugar-Sweetened Beverages Consumption | Highest vs. lowest | CD | 1 | Khademi2021 | NA/1,987^a,b^ | RR | 1.22, 0.91-1.64 | 4/0/1/3 | Random | 75.2;0.003 | NA | 8 | NS |
| Sugar-Sweetened Beverages Consumption | Highest vs. lowest | IBD | 1 | Khademi2021 | NA/1,622^a,b^ | RR | 1.02, 0.94-1.11 | 3/0/1/2 | Random | 19.4;0.29 | NA | 8 | NS |
| Tetanus vaccination | Yes vs. no | IBD | 1 | Pineton2015 | NA/524^b^ | RR | 1.27, 0.77-208 | 3/0/0/3 | Random | 0;0.7096 | NA | 8 | NS |
| Tetanus vaccination | Yes vs. no | IBD | 1 | Pineton2015 | NA/1,255^b^ | RR | 1.07, 0.58-1.98 | 3/0/0/3 | Random | 78；0.0107 | NA | 8 | NS |
| Tonsillectomy | Yes vs. no | UCwest | 2 | Zhao2021 | NA/59,130^a,b^ | OR | 0.99, 0.86-1.15 | 15/0/1/14 | Random | 64.9;0 | 0.028 | 8 | NS |
| Tonsillectomy | Yes vs. no | UCeast | 2 | Zhao2021 | NA/1,151^b^ | OR | 1.17, 0.79-1.73 | 5/0/0/5 | Random | 39;0.161 | 0.028 | 8 | NS |
| Tonsillectomy | Yes vs. no | CDeast | 2 | Zhao2021 | NA/662^b^ | OR | 1.80, 1.00-3.24 | 5/0/0/5 | Random | 48;0.104 | 0.39 | 8 | NS |
| Total carbohydrates intake | Highest vs. lowest | UC | 6 | Khademi2021 | NA/969^a,b^ | RR | 1.22, 0.70-2.14 | 4/0/3/1 | Random | 45.2;0.12 | NA | 8 | NS |
| Total carbohydrates intake | Highest vs. lowest | CD | 6 | Khademi2021 | NA/969^a,b^ | RR | 1.06, 0.64-1.75 | 4/0/3/1 | Random | 7.2;0.36 | NA | 8 | NS |
| Total carbohydrates intake | Highest vs. lowest | IBD(UC/CD) | 6 | Khademi2021 | NA/969^a,b^ | RR | 1.16, 0.78-1.73 | 4/0/3/1 | Random | 36.4；0.17 | NA | 8 | NS |
| Total energy | Highest vs. lowest | UCwest | 1 | Zhao2021 | NA/461^b^ | OR | 1.23, 1.00-1.50 | 3/0/0/3 | Random | 14.8;0.303 | NA | 8 | NS |
| Urban living | Yes vs. no | CDeast | 4 | Zhao2021 | NA/688^b^ | OR | 1.29, 0.79-2.13 | 3/0/0/3 | Random | 82.1;0.001 | 0.071 | 8 | NS |
| Urban living | Yes vs. no | UCeast | 4 | Zhao2021 | NA/2,306^b^ | OR | 0.86, 0.71-1.04 | 4/0/0/4 | Random | 0;0.773 | 0.796 | 8 | NS |
| Vegetables | Highest vs. lowest | CDeast | 5 | Zhao2021 | NA/943^b^ | OR | 1.01, 0.72-1.41 | 8/0/0/8 | Random | 54.4;0.019 | 0.041 | 8 | NS |
| Vegetables | Highest vs. lowest | UCwest | 5 | Zhao2021 | NA/1,023^a,b^ | OR | 0.89, 0.77-1.04 | 8/0/2/6 | Random | 39.5;0064 | 0.778 | 8 | NS |
| Vegetables | Highest vs. lowest | UCeast | 5 | Zhao2021 | NA/2,855^b^ | OR | 0.80, 0.58-1.10 | 10/0/0/10 | Random | 81.7;0 | 0.778 | 8 | NS |
| Western/unhealthy dietary patterns | Yes vs. no | UC | 1 | Khorshidi2020 | NA^a,b^ | OR | 0.97, 0.67-1.26 | 3/0/1/2 | fixed | 0;0.591 | 0.091 | 8 | NS |
| Western/unhealthy dietary patterns | Yes vs. no | CD | 1 | Khorshidi2020 | NA^a,b^ | OR | 0.78, 0.51-1.04 | 6/0/2/4 | fixed | 31.3;0.201 | 91 | 8 | NS |
| α-Linolenic acid | Highest vs. lowest | IBD | 1 | Mozaffari2020 | NA/868^a,b^ | ES | 1.17, 0.63-2.17 | 3/0/2/1 | Fixed | 0; 0.40 | NA | 8 | NS |

Notes: CD: Crohn’s disease; UC: ulcerative colitis; IBD: Inflammatory bowel disease; BMI: Body Mass Index; HCMV: human cytomegalovirus; MUFA: monounsaturated fatty acid; PUFA: polyunstatured fatty acid; BGG: Bacillus Calmette Guerin; EHS: Enterohepatic Helicobacter Species; NA: Not Avaliable; OR: Odds ratio; RR: Relative risk; HR: Hazard ratio; ES: Effect Sizes; pOR: pooled OR

a. Cohort;

b. Case-control;

c. Unclear

**Supplementary Table S2.** Assessments of AMSTAR scores

| Risk factors | Outcome | Study | A priori design provided | Duplicate study selection & data extraction | At least two electronic databases searched | Status of  publication used as an inclusion criterion | List of  included and excluded studies provided | Characteristics of included  studies provided | Scientific quality of  included studies assessed | Scientific quality of the included studies used  appropriately to form  conclusions | Appropriate methods to  combine studies | Publication bias assessed | Conflict of interest included | Total AMSTAR Score |
| --- | --- | --- | --- | --- | --- | --- | --- | --- | --- | --- | --- | --- | --- | --- |
| Antibiotic use | CDwest | Zhao2021 | 0 | 1 | 1 | 0 | 1 | 1 | 1 | 0 | 1 | 1 | 1 | 8 |
| Antibiotic use | UCwest | Zhao2021 | 0 | 1 | 1 | 0 | 1 | 1 | 1 | 0 | 1 | 1 | 1 | 8 |
| Appendectomy | CD | Zhang2023 | 1 | 1 | 1 | 0 | 1 | 1 | 1 | 0 | 1 | 1 | 1 | 9 |
| Asthma | CD | Kuenzig2018 | 1 | 1 | 1 | 0 | 1 | 1 | 1 | 0 | 1 | 1 | 1 | 9 |
| Asthma | UC | Kuenzig2018 | 1 | 1 | 1 | 0 | 1 | 1 | 1 | 0 | 1 | 1 | 1 | 9 |
| Atopic dermatitis | UC | Lee 2020 | 0 | 1 | 1 | 0 | 1 | 1 | 1 | 0 | 1 | 1 | 1 | 8 |
| Autism spectrum disorder | IBD | Kim2022 | 1 | 1 | 1 | 0 | 1 | 1 | 1 | 0 | 1 | 1 | 1 | 9 |
| Bariatric Surgery | IBD | Kermansaravi2022 | 1 | 1 | 1 | 0 | 1 | 1 | 1 | 0 | 1 | 1 | 1 | 9 |
| BMI | IBD | Milajerdi2022 | 0 | 1 | 1 | 0 | 1 | 1 | 1 | 0 | 1 | 1 | 1 | 8 |
| Breast feeding | CDwest | Zhao2021 | 0 | 1 | 1 | 0 | 1 | 1 | 1 | 0 | 1 | 1 | 1 | 8 |
| Breast feeding | CDeast | Zhao2021 | 0 | 1 | 1 | 0 | 1 | 1 | 1 | 0 | 1 | 1 | 1 | 8 |
| Campylobacter species | IBD | Castaño-Rodríguez2017 | 0 | 0 | 1 | 0 | 1 | 1 | 1 | 0 | 1 | 1 | 1 | 7 |
| Ceasarean | CDwest | Zhao2021 | 0 | 1 | 1 | 0 | 1 | 1 | 1 | 0 | 1 | 1 | 1 | 8 |
| Ceasarean | UCwest | Zhao2021 | 0 | 1 | 1 | 0 | 1 | 1 | 1 | 0 | 1 | 1 | 1 | 8 |
| Cholesterol | UCeast | Zhao2021 | 0 | 1 | 1 | 0 | 1 | 1 | 1 | 0 | 1 | 1 | 1 | 8 |
| Chronic obstructive pulmonary diseases | CD | Labarca2019 | 1 | 1 | 1 | 0 | 1 | 1 | 1 | 1 | 1 | 1 | 1 | 10 |
| Chronic obstructive pulmonary diseases | UC | Labarca2019 | 1 | 1 | 1 | 0 | 1 | 1 | 1 | 1 | 1 | 1 | 1 | 10 |
| Chronic obstructive pulmonary diseases | CD/UC | Labarca2019 | 1 | 1 | 1 | 0 | 1 | 1 | 1 | 1 | 1 | 1 | 1 | 10 |
| Coffee | UCwest | Zhao2021 | 0 | 1 | 1 | 0 | 1 | 1 | 1 | 0 | 1 | 1 | 1 | 8 |
| Coffee | UCeast | Zhao2021 | 0 | 1 | 1 | 0 | 1 | 1 | 1 | 0 | 1 | 1 | 1 | 8 |
| Contact with farm animals | CDwest | Zhao2021 | 0 | 1 | 1 | 0 | 1 | 1 | 1 | 0 | 1 | 1 | 1 | 8 |
| Dietary fat | IBD,UC,CD | Wu2016 | 0 | 1 | 1 | 0 | 1 | 1 | 1 | 0 | 1 | 1 | 1 | 8 |
| Dietary fiber intake | IBD | Milajerdi2021 | 0 | 1 | 1 | 0 | 1 | 1 | 1 | 0 | 1 | 1 | 1 | 8 |
| Dietary fiber intake | CD | Milaherdi2021 | 0 | 1 | 1 | 0 | 1 | 1 | 1 | 0 | 1 | 1 | 1 | 8 |
| Dietary fiber intake | CD | Milaherdi2021 | 0 | 1 | 1 | 0 | 1 | 1 | 1 | 0 | 1 | 1 | 1 | 8 |
| Egg | UCeast | Zhao2021 | 0 | 1 | 1 | 0 | 1 | 1 | 1 | 0 | 1 | 1 | 1 | 8 |
| Exposure to pets | CDeast | Zhao2021 | 0 | 1 | 1 | 0 | 1 | 1 | 1 | 0 | 1 | 1 | 1 | 8 |
| Exposure to pets | UCwest | Zhao2021 | 0 | 1 | 1 | 0 | 1 | 1 | 1 | 0 | 1 | 1 | 1 | 8 |
| Exposure to pets | UCeast | Zhao2021 | 0 | 1 | 1 | 0 | 1 | 1 | 1 | 0 | 1 | 1 | 1 | 8 |
| Exposure to pets | CDwest | Zhao2021 | 0 | 1 | 1 | 0 | 1 | 1 | 1 | 0 | 1 | 1 | 1 | 8 |
| Fatty acids | UCeast | Zhao2021 | 0 | 1 | 1 | 0 | 1 | 1 | 1 | 0 | 1 | 1 | 1 | 8 |
| Fiber intake | CD | Zeng2017 | 0 | 1 | 1 | 0 | 1 | 1 | 1 | 0 | 1 | 1 | 1 | 8 |
| Fruit | UC | Milajerdi2021 | 0 | 1 | 1 | 0 | 1 | 1 | 1 | 0 | 1 | 1 | 1 | 8 |
| Fruit | CD | Milajerdi2021 | 0 | 1 | 1 | 0 | 1 | 1 | 1 | 0 | 1 | 1 | 1 | 8 |
| Fruit | IBD | Milajerdi2021 | 0 | 1 | 1 | 0 | 1 | 1 | 1 | 0 | 1 | 1 | 1 | 8 |
| HCMV infection | I BD | Lv2017 | 0 | 1 | 1 | 0 | 1 | 1 | 1 | 0 | 1 | 1 | 1 | 8 |
| Healthy/prudent dietary patterns | CD | Khorshidi2020 | 0 | 1 | 1 | 0 | 1 | 1 | 1 | 0 | 1 | 1 | 1 | 8 |
| Helicobacter pylori | IBD | Bouriat2022 | 0 | 1 | 1 | 0 | 1 | 1 | 1 | 0 | 1 | 1 | 1 | 8 |
| Hidradenitis suppurativa | CD | Phan2020 | 1 | 1 | 1 | 0 | 1 | 1 | 1 | 1 | 1 | 1 | 1 | 10 |
| Hidradenitis suppurativa | UC | Phan2020 | 1 | 1 | 1 | 0 | 1 | 1 | 1 | 1 | 1 | 1 | 1 | 10 |
| Hidradenitis suppurativa | Unspecified IBD | Phan2020 | 1 | 1 | 1 | 0 | 1 | 1 | 1 | 1 | 1 | 1 | 1 | 10 |
| IBD family history | UCwest | Zhao2021 | 0 | 1 | 1 | 0 | 1 | 1 | 1 | 0 | 1 | 1 | 1 | 8 |
| IBD family history | UCeast | Zhao2021 | 0 | 1 | 1 | 0 | 1 | 1 | 1 | 0 | 1 | 1 | 1 | 8 |
| IBD family history | CDwest | Zhao2021 | 0 | 1 | 1 | 0 | 1 | 1 | 1 | 0 | 1 | 1 | 1 | 8 |
| IBD family history | CDeast | Zhao2021 | 0 | 1 | 1 | 0 | 1 | 1 | 1 | 0 | 1 | 1 | 1 | 8 |
| Isotretinoin | UCwest | Zhao2021 | 0 | 1 | 1 | 0 | 1 | 1 | 1 | 0 | 1 | 1 | 1 | 8 |
| Meat and meat product | CDwest | Zhao2021 | 0 | 1 | 1 | 0 | 1 | 1 | 1 | 0 | 1 | 1 | 1 | 8 |
| Milk and dairy products | CDwest | Zhao2021 | 0 | 1 | 1 | 0 | 1 | 1 | 1 | 0 | 1 | 1 | 1 | 8 |
| More physical activity | CDwest | Zhao2021 | 0 | 1 | 1 | 0 | 1 | 1 | 1 | 0 | 1 | 1 | 1 | 8 |
| More physical activity | CDeast | Zhao2021 | 0 | 1 | 1 | 0 | 1 | 1 | 1 | 0 | 1 | 1 | 1 | 8 |
| More physical activity | UCeast | Zhao2021 | 0 | 1 | 1 | 0 | 1 | 1 | 1 | 0 | 1 | 1 | 1 | 8 |
| MUFA | UCeast | Zhao2021 | 0 | 1 | 1 | 0 | 1 | 1 | 1 | 0 | 1 | 1 | 1 | 8 |
| Multiple birth | CDwest | Zhao2021 | 0 | 1 | 1 | 0 | 1 | 1 | 1 | 0 | 1 | 1 | 1 | 8 |
| Multiple birth | UCeast | Zhao2021 | 0 | 1 | 1 | 0 | 1 | 1 | 1 | 0 | 1 | 1 | 1 | 8 |
| Multiple sclerosis | UC | Wang2022 | 0 | 1 | 1 | 0 | 1 | 1 | 1 | 0 | 1 | 1 | 1 | 8 |
| Multiple sclerosis | CD | Wang2022 | 0 | 1 | 1 | 0 | 1 | 1 | 1 | 0 | 1 | 1 | 1 | 8 |
| Multiple sclerosis | UC and CD | Wang2022 | 0 | 1 | 1 | 0 | 1 | 1 | 1 | 0 | 1 | 1 | 1 | 8 |
| n-6PUFA | UCwest | Zhao2021 | 0 | 1 | 1 | 0 | 1 | 1 | 1 | 0 | 1 | 1 | 1 | 8 |
| Number of siblings | CD | Cholapranee2016 | 0 | 1 | 1 | 0 | 1 | 1 | 1 | 0 | 1 | 1 | 1 | 8 |
| Oral contraceptive | CDwest | Zhao2021 | 0 | 1 | 1 | 0 | 1 | 1 | 1 | 0 | 1 | 1 | 1 | 8 |
| Oral contraceptive | UCweat | Zhao2021 | 0 | 1 | 1 | 0 | 1 | 1 | 1 | 0 | 1 | 1 | 1 | 8 |
| Otitis media infection | IBD | Agrawal2021 | 1 | 1 | 1 | 0 | 1 | 1 | 1 | 0 | 1 | 1 | 1 | 9 |
| Otitis media infection | CD | Agrawal2021 | 1 | 1 | 1 | 0 | 1 | 1 | 1 | 0 | 1 | 1 | 1 | 9 |
| Periodontal | IBD | Lorenzo-Pouso2021 | 0 | 1 | 1 | 0 | 1 | 1 | 1 | 1 | 1 | 1 | 1 | 9 |
| Periodontal | CD | Lorenzo-Pouso2021 | 0 | 1 | 1 | 0 | 1 | 1 | 1 | 1 | 1 | 1 | 1 | 9 |
| Periodontal | UC | Lorenzo-Pouso2021 | 0 | 1 | 1 | 0 | 1 | 1 | 1 | 1 | 1 | 1 | 1 | 9 |
| Personal toilet | UC | Cholapranee2016 | 0 | 1 | 1 | 0 | 1 | 1 | 1 | 0 | 1 | 1 | 1 | 8 |
| Poliomyelitis | CD | Pineton2015 | 0 | 1 | 1 | 0 | 1 | 1 | 1 | 0 | 1 | 1 | 1 | 8 |
| Poliomyelitis | UC | Pineton2015 | 0 | 1 | 1 | 0 | 1 | 1 | 1 | 0 | 1 | 1 | 1 | 8 |
| Proton pump inhibitors, PPIs | IBD | Shastri2022 | 1 | 1 | 1 | 0 | 1 | 1 | 1 | 0 | 1 | 1 | 1 | 9 |
| Psoriasis | CD | Fu2018 | 0 | 1 | 1 | 0 | 1 | 1 | 1 | 0 | 1 | 1 | 1 | 8 |
| Psoriasis | UC | Fu2018 | 0 | 1 | 1 | 0 | 1 | 1 | 1 | 0 | 1 | 1 | 1 | 8 |
| Rosacea | IBD | Han2019 | 0 | 1 | 1 | 0 | 1 | 1 | 1 | 0 | 1 | 1 | 1 | 8 |
| Smoking | CDwest | Zhao2021 | 0 | 1 | 1 | 0 | 1 | 1 | 1 | 0 | 1 | 1 | 1 | 8 |
| Smoking | CDeast | Zhao2021 | 0 | 1 | 1 | 0 | 1 | 1 | 1 | 0 | 1 | 1 | 1 | 8 |
| Soft drink | CDwest | Zhao2021 | 0 | 1 | 1 | 0 | 1 | 1 | 1 | 0 | 1 | 1 | 1 | 8 |
| Suger | UC | Khademi2021 | 0 | 1 | 1 | 0 | 1 | 1 | 1 | 0 | 1 | 1 | 1 | 8 |
| Suger | CD | Khademi2021 | 0 | 1 | 1 | 0 | 1 | 1 | 1 | 0 | 1 | 1 | 1 | 8 |
| Suger | IBD | Khademi2021 | 0 | 1 | 1 | 0 | 1 | 1 | 1 | 0 | 1 | 1 | 1 | 8 |
| Tea consumption | CD | Yang2019 | 0 | 1 | 1 | 0 | 1 | 1 | 1 | 0 | 1 | 1 | 1 | 8 |
| Tea consumption | UC | Nie2017 | 0 | 1 | 1 | 0 | 1 | 1 | 1 | 0 | 1 | 1 | 1 | 8 |
| Tonsillectomy | CDwest | Zhao2021 | 0 | 1 | 1 | 0 | 1 | 1 | 1 | 0 | 1 | 1 | 1 | 8 |
| Total energy | CDwest | Zhao2021 | 0 | 1 | 1 | 0 | 1 | 1 | 1 | 0 | 1 | 1 | 1 | 8 |
| Urban living | CDwest | Zhao2021 | 0 | 1 | 1 | 0 | 1 | 1 | 1 | 0 | 1 | 1 | 1 | 8 |
| Urban living | UCwest | Zhao2021 | 0 | 1 | 1 | 0 | 1 | 1 | 1 | 0 | 1 | 1 | 1 | 8 |
| Vegetables | CDwest | Zhao2021 | 0 | 1 | 1 | 0 | 1 | 1 | 1 | 0 | 1 | 1 | 1 | 8 |
| Vitamin D | CDwest | Zhao2021 | 0 | 1 | 1 | 0 | 1 | 1 | 1 | 0 | 1 | 1 | 1 | 8 |
| Vitamin D | UCwest | Zhao2021 | 0 | 1 | 1 | 0 | 1 | 1 | 1 | 0 | 1 | 1 | 1 | 8 |
| Vitamin D | UCeast | Zhao2021 | 0 | 1 | 1 | 0 | 1 | 1 | 1 | 0 | 1 | 1 | 1 | 8 |
| Western dietary pattern | CD | Li2020 | 0 | 1 | 1 | 0 | 1 | 1 | 1 | 0 | 1 | 1 | 1 | 8 |
| Western dietary pattern | UC | Li2020 | 0 | 1 | 1 | 0 | 1 | 1 | 1 | 0 | 1 | 1 | 1 | 8 |
| Western dietary pattern | IBDCD and UC | Li2020 | 0 | 1 | 1 | 0 | 1 | 1 | 1 | 0 | 1 | 1 | 1 | 8 |
| 35older maternal age ≥35 years | IBD | Agrawal2021 | 1 | 1 | 1 | 0 | 1 | 1 | 1 | 0 | 1 | 1 | 1 | 9 |
| 35older maternal age ≥35 years | CD | Agrawal2021 | 1 | 1 | 1 | 0 | 1 | 1 | 1 | 0 | 1 | 1 | 1 | 9 |
| Alcohol | UC | Nie2017 | 0 | 1 | 1 | 0 | 1 | 1 | 1 | 0 | 1 | 1 | 1 | 8 |
| Alcohol | CD | Yang2019 | 0 | 1 | 1 | 0 | 1 | 1 | 1 | 0 | 1 | 1 | 1 | 8 |
| Antibiotic use | CDeast | Zhao2021 | 0 | 1 | 1 | 0 | 1 | 1 | 1 | 0 | 1 | 1 | 1 | 8 |
| antibiotic use | UCeast | Zhao2021 | 0 | 1 | 1 | 0 | 1 | 1 | 1 | 0 | 1 | 1 | 1 | 8 |
| Atopic dermatitis | CD | Lee 2020 | 0 | 1 | 1 | 0 | 1 | 1 | 1 | 0 | 1 | 1 | 1 | 8 |
| Bed or bedroom sharing | UCwest | Zhao2021 | 0 | 1 | 1 | 0 | 1 | 1 | 1 | 0 | 1 | 1 | 1 | 8 |
| Bed or bedroom sharing | UCeast | Zhao2021 | 0 | 1 | 1 | 0 | 1 | 1 | 1 | 0 | 1 | 1 | 1 | 8 |
| Bed or bedroom sharing | CDwest | Zhao2021 | 0 | 1 | 1 | 0 | 1 | 1 | 1 | 0 | 1 | 1 | 1 | 8 |
| BGG vaccine | IBD | Agrawal2021 | 1 | 1 | 1 | 0 | 1 | 1 | 1 | 0 | 1 | 1 | 1 | 9 |
| BGG vaccine | CD | Agrawal2021 | 1 | 1 | 1 | 0 | 1 | 1 | 1 | 0 | 1 | 1 | 1 | 9 |
| BMI | CD | Milajerdi2022 | 0 | 1 | 1 | 0 | 1 | 1 | 1 | 0 | 1 | 1 | 1 | 8 |
| Coffee | CDwest | Zhao2021 | 0 | 1 | 1 | 0 | 1 | 1 | 1 | 0 | 1 | 1 | 1 | 8 |
| Dietary fiber intake | UC | Milaherdi2021 | 0 | 1 | 1 | 0 | 1 | 1 | 1 | 0 | 1 | 1 | 1 | 8 |
| Diphteria and tetanus containing vaccines | IBD | Pineton2015 | 0 | 1 | 1 | 0 | 1 | 1 | 1 | 0 | 1 | 1 | 1 | 8 |
| Egg | CDwest | Zhao2021 | 0 | 1 | 1 | 0 | 1 | 1 | 1 | 0 | 1 | 1 | 1 | 8 |
| Egg | CDeast | Zhao2021 | 0 | 1 | 1 | 0 | 1 | 1 | 1 | 0 | 1 | 1 | 1 | 8 |
| EHS | IBD | Castaño-Rodríguez2017 | 0 | 0 | 1 | 0 | 1 | 1 | 1 | 0 | 1 | 1 | 1 | 7 |
| Exposure to pets | CDWest | Zhao2021 | 0 | 1 | 1 | 0 | 1 | 1 | 1 | 0 | 1 | 1 | 1 | 8 |
| Fat | CDwest | Zhao2021 | 0 | 1 | 1 | 0 | 1 | 1 | 1 | 0 | 1 | 1 | 1 | 8 |
| Fat | UCwest | Zhao2021 | 0 | 1 | 1 | 0 | 1 | 1 | 1 | 0 | 1 | 1 | 1 | 8 |
| Fat | UCeast | Zhao2021 | 0 | 1 | 1 | 0 | 1 | 1 | 1 | 0 | 1 | 1 | 1 | 8 |
| Fatty acids | CDwest | Zhao2021 | 0 | 1 | 1 | 0 | 1 | 1 | 1 | 0 | 1 | 1 | 1 | 8 |
| Fatty acids | UCwest | Zhao2021 | 0 | 1 | 1 | 0 | 1 | 1 | 1 | 0 | 1 | 1 | 1 | 8 |
| Fiber intake | UC | Wang2017 | 0 | 1 | 1 | 0 | 1 | 1 | 1 | 0 | 1 | 1 | 1 | 8 |
| Fish | IBD | Mozaffari2020 | 0 | 1 | 1 | 0 | 1 | 1 | 1 | 0 | 1 | 1 | 1 | 8 |
| Fruits | UCwest | Zhao2021 | 0 | 1 | 1 | 0 | 1 | 1 | 1 | 0 | 1 | 1 | 1 | 8 |
| Hot water | CD | Cholapranee2016 | 0 | 1 | 1 | 0 | 1 | 1 | 1 | 0 | 1 | 1 | 1 | 8 |
| Hot water | UC | Cholapranee2016 | 0 | 1 | 1 | 0 | 1 | 1 | 1 | 0 | 1 | 1 | 1 | 8 |
| Isotretinoin | CDwest | Zhao2021 | 0 | 1 | 1 | 0 | 1 | 1 | 1 | 0 | 1 | 1 | 1 | 8 |
| Job strain | CD | Heikkilä2014 | 0 | 1 | 1 | 0 | 1 | 1 | 1 | 0 | 1 | 1 | 1 | 8 |
| Job strain | UC | Heikkilä2014 | 0 | 1 | 1 | 0 | 1 | 1 | 1 | 0 | 1 | 1 | 1 | 8 |
| Low birth weight | IBD | Agrawal2021 | 1 | 1 | 1 | 0 | 1 | 1 | 1 | 0 | 1 | 1 | 1 | 9 |
| Low birth weight | CD | Agrawal2021 | 1 | 1 | 1 | 0 | 1 | 1 | 1 | 0 | 1 | 1 | 1 | 9 |
| Low birth weight | UC | Agrawal2021 | 1 | 1 | 1 | 0 | 1 | 1 | 1 | 0 | 1 | 1 | 1 | 9 |
| Measles vaccine | IBD | Agrawal2021 | 1 | 1 | 1 | 0 | 1 | 1 | 1 | 0 | 1 | 1 | 1 | 9 |
| Measles vaccine | CD | Agrawal2021 | 1 | 1 | 1 | 0 | 1 | 1 | 1 | 0 | 1 | 1 | 1 | 9 |
| Measles vaccine | UC | Agrawal2021 | 1 | 1 | 1 | 0 | 1 | 1 | 1 | 0 | 1 | 1 | 1 | 9 |
| Meat and meat product | UCwest | Zhao2021 | 0 | 1 | 1 | 0 | 1 | 1 | 1 | 0 | 1 | 1 | 1 | 8 |
| Meat and meat product | UCeast | Zhao2021 | 0 | 1 | 1 | 0 | 1 | 1 | 1 | 0 | 1 | 1 | 1 | 8 |
| Milk and dairy products | CDeast | Zhao2021 | 0 | 1 | 1 | 0 | 1 | 1 | 1 | 0 | 1 | 1 | 1 | 8 |
| Milk and dairy products | UCeast | Zhao2021 | 0 | 1 | 1 | 0 | 1 | 1 | 1 | 0 | 1 | 1 | 1 | 8 |
| Milk and dairy products | UCwest | Zhao2021 | 0 | 1 | 1 | 0 | 1 | 1 | 1 | 0 | 1 | 1 | 1 | 8 |
| More physical activity | UCwest | Zhao2021 | 0 | 1 | 1 | 0 | 1 | 1 | 1 | 0 | 1 | 1 | 1 | 8 |
| MUFA | CDwest | Zhao2021 | 0 | 1 | 1 | 0 | 1 | 1 | 1 | 0 | 1 | 1 | 1 | 8 |
| MUFA | UCwest | Zhao2021 | 0 | 1 | 1 | 0 | 1 | 1 | 1 | 0 | 1 | 1 | 1 | 8 |
| Multiple birth | UCwest | Zhao2021 | 0 | 1 | 1 | 0 | 1 | 1 | 1 | 0 | 1 | 1 | 1 | 8 |
| n-3PUFAs | IBD | Mozaffari2020 | 0 | 1 | 1 | 0 | 1 | 1 | 1 | 0 | 1 | 1 | 1 | 8 |
| n-3PUFAs | UC | Mozaffari2020 | 0 | 1 | 1 | 0 | 1 | 1 | 1 | 0 | 1 | 1 | 1 | 8 |
| n-3PUFAs | CD | Mozaffari2020 | 0 | 1 | 1 | 0 | 1 | 1 | 1 | 0 | 1 | 1 | 1 | 8 |
| n-6PUFA | CDwest | Zhao2021 | 0 | 1 | 1 | 0 | 1 | 1 | 1 | 0 | 1 | 1 | 1 | 8 |
| n-6PUFA | UCeast | Zhao2021 | 0 | 1 | 1 | 0 | 1 | 1 | 1 | 0 | 1 | 1 | 1 | 8 |
| Number of siblings | UC | Cholapranee2016 | 0 | 1 | 1 | 0 | 1 | 1 | 1 | 0 | 1 | 1 | 1 | 8 |
| Number of siblings | IBD | Cholapranee2016 | 0 | 1 | 1 | 0 | 1 | 1 | 1 | 0 | 1 | 1 | 1 | 8 |
| Older Maternal age≥35 years | IBD | Agrawal2021 | 1 | 1 | 1 | 0 | 1 | 1 | 1 | 0 | 1 | 1 | 1 | 9 |
| Older Maternal age≥35 years | CD | Agrawal2021 | 1 | 1 | 1 | 0 | 1 | 1 | 1 | 0 | 1 | 1 | 1 | 9 |
| Oral contraceptive | CDeast | Zhao2021 | 0 | 1 | 1 | 0 | 1 | 1 | 1 | 0 | 1 | 1 | 1 | 8 |
| Oral contraceptive | UCeast | Zhao2021 | 0 | 1 | 1 | 0 | 1 | 1 | 1 | 0 | 1 | 1 | 1 | 8 |
| Passive exposure to tobacco smoke | CD | Agrawal2021 | 1 | 1 | 1 | 0 | 1 | 1 | 1 | 0 | 1 | 1 | 1 | 9 |
| Personal toilet | CD | Cholapranee2016 | 0 | 1 | 1 | 0 | 1 | 1 | 1 | 0 | 1 | 1 | 1 | 8 |
| Poliomyelitis | IBD | Pineton2015 | 0 | 1 | 1 | 0 | 1 | 1 | 1 | 0 | 1 | 1 | 1 | 8 |
| Premature birth | IBD | Agrawal2021 | 1 | 1 | 1 | 0 | 1 | 1 | 1 | 0 | 1 | 1 | 1 | 9 |
| Premature birth | CD | Agrawal2021 | 1 | 1 | 1 | 0 | 1 | 1 | 1 | 0 | 1 | 1 | 1 | 9 |
| Premature birth | UC | Agrawal2021 | 1 | 1 | 1 | 0 | 1 | 1 | 1 | 0 | 1 | 1 | 1 | 9 |
| Premature birth | IBD | Agrawal2021 | 1 | 1 | 1 | 0 | 1 | 1 | 1 | 0 | 1 | 1 | 1 | 9 |
| Premature birth | CD | Agrawal2021 | 1 | 1 | 1 | 0 | 1 | 1 | 1 | 0 | 1 | 1 | 1 | 9 |
| Premature birth | UC | Agrawal2021 | 1 | 1 | 1 | 0 | 1 | 1 | 1 | 0 | 1 | 1 | 1 | 9 |
| Primary sclerosing cholangitis | IBD | Zhang2022 | 0 | 1 | 1 | 0 | 1 | 1 | 1 | 0 | 1 | 1 | 1 | 8 |
| Protein intake | UC/IBD | Zhou2022 | 0 | 1 | 1 | 0 | 1 | 1 | 1 | 0 | 1 | 1 | 1 | 8 |
| PUFAs Polyunsaturated fat intake | UC | Wang2016 | 0 | 1 | 1 | 0 | 1 | 1 | 1 | 0 | 1 | 1 | 1 | 8 |
| Smoking | UCwest | Zhao2021 | 0 | 1 | 1 | 0 | 1 | 1 | 1 | 0 | 1 | 1 | 1 | 8 |
| Smoking | UCeast | Zhao2021 | 0 | 1 | 1 | 0 | 1 | 1 | 1 | 0 | 1 | 1 | 1 | 8 |
| Soft drink | CDeast | Zhao2021 | 0 | 1 | 1 | 0 | 1 | 1 | 1 | 0 | 1 | 1 | 1 | 8 |
| Soft drink | UCwest | Zhao2021 | 0 | 1 | 1 | 0 | 1 | 1 | 1 | 0 | 1 | 1 | 1 | 8 |
| Soft drink | UCeast | Zhao2021 | 0 | 1 | 1 | 0 | 1 | 1 | 1 | 0 | 1 | 1 | 1 | 8 |
| Statin use | IBD | Bhagavathula2021 | 0 | 1 | 1 | 0 | 1 | 1 | 1 | 0 | 1 | 1 | 1 | 8 |
| Statin use | CD | Bhagavathula2021 | 0 | 1 | 1 | 0 | 1 | 1 | 1 | 0 | 1 | 1 | 1 | 8 |
| Statin use | UC | Bhagavathula2021 | 0 | 1 | 1 | 0 | 1 | 1 | 1 | 0 | 1 | 1 | 1 | 8 |
| Sugar-Sweetened Beverages Consumption | UC | Khademi2021 | 0 | 1 | 1 | 0 | 1 | 1 | 1 | 0 | 1 | 1 | 1 | 8 |
| Sugar-Sweetened Beverages Consumption | CD | Khademi2021 | 0 | 1 | 1 | 0 | 1 | 1 | 1 | 0 | 1 | 1 | 1 | 8 |
| Sugar-Sweetened Beverages Consumption | IBD | Khademi2021 | 0 | 1 | 1 | 0 | 1 | 1 | 1 | 0 | 1 | 1 | 1 | 8 |
| Tetanus vaccination | IBD | Pineton2015 | 0 | 1 | 1 | 0 | 1 | 1 | 1 | 0 | 1 | 1 | 1 | 8 |
| Tetanus vaccination | IBD | Pineton2015 | 0 | 1 | 1 | 0 | 1 | 1 | 1 | 0 | 1 | 1 | 1 | 8 |
| Tonsillectomy | UCwest | Zhao2021 | 0 | 1 | 1 | 0 | 1 | 1 | 1 | 0 | 1 | 1 | 1 | 8 |
| Tonsillectomy | UCeast | Zhao2021 | 0 | 1 | 1 | 0 | 1 | 1 | 1 | 0 | 1 | 1 | 1 | 8 |
| Tonsillectomy | CDeast | Zhao2021 | 0 | 1 | 1 | 0 | 1 | 1 | 1 | 0 | 1 | 1 | 1 | 8 |
| Total carbohydrates intake | UC | Khademi2021 | 0 | 1 | 1 | 0 | 1 | 1 | 1 | 0 | 1 | 1 | 1 | 8 |
| Total carbohydrates intake | CD | Khademi2021 | 0 | 1 | 1 | 0 | 1 | 1 | 1 | 0 | 1 | 1 | 1 | 8 |
| Total carbohydrates intake | IBD(UC/CD) | Khademi2021 | 0 | 1 | 1 | 0 | 1 | 1 | 1 | 0 | 1 | 1 | 1 | 8 |
| Total energy | UCwest | Zhao2021 | 0 | 1 | 1 | 0 | 1 | 1 | 1 | 0 | 1 | 1 | 1 | 8 |
| Urban living | CDeast | Zhao2021 | 0 | 1 | 1 | 0 | 1 | 1 | 1 | 0 | 1 | 1 | 1 | 8 |
| Urban living | UCeast | Zhao2021 | 0 | 1 | 1 | 0 | 1 | 1 | 1 | 0 | 1 | 1 | 1 | 8 |
| Vegetables | CDeast | Zhao2021 | 0 | 1 | 1 | 0 | 1 | 1 | 1 | 0 | 1 | 1 | 1 | 8 |
| Vegetables | UCwest | Zhao2021 | 0 | 1 | 1 | 0 | 1 | 1 | 1 | 0 | 1 | 1 | 1 | 8 |
| Vegetables | UCeast | Zhao2021 | 0 | 1 | 1 | 0 | 1 | 1 | 1 | 0 | 1 | 1 | 1 | 8 |
| Western/unhealthy dietary patterns | UC | Khorshidi2020 | 0 | 1 | 1 | 0 | 1 | 1 | 1 | 0 | 1 | 1 | 1 | 8 |
| Western/unhealthy dietary patterns | CD | Khorshidi2020 | 0 | 1 | 1 | 0 | 1 | 1 | 1 | 0 | 1 | 1 | 1 | 8 |
| α-Linolenic acid | IBD | Mozaffari2020 | 0 | 1 | 1 | 0 | 1 | 1 | 1 | 0 | 1 | 1 | 1 | 8 |

**Notes:** CD: Crohn’s disease; UC: ulcerative colitis; IBD: Inflammatory bowel disease; BMI: Body Mass Index; HCMV: human cytomegalovirus; MUFA: monounsaturated fatty acid; PUFA: polyunstatured fatty acid; BGG: Bacillus Calmette Guerin; EHS: Enterohepatic Helicobacter Species; NA: Not Avaliable; OR: Odds ratio; RR: Relative risk; HR: Hazard ratio; ES: Effect Sizes; pOR: pooled OR.
